# General Index to the British and Foreign Medical Review

**Published:** 1848

**Authors:** 


Rilliet and Barthez, on diseases
of children . . xvii 293, 312
Rilliet, M., on diseases of children xii 350
Ring fast on finger, mode of removing vii 515
Ringworm, nature of . . . vi 429
tincture of iodine in . . viii 591
Riofrey, Dr., on female education v 380, 405
Rio Medical Review . . . ii 234
Rita-Cristina, a double female . viii 34
the monster . . . . xii 379
"Rival Discoverers, the" . . v 621
Rivalry and jealousies of medical men xxi 446
Rix, Mr., Dr. Armstrong's lectures by i 34
Roaring disease, in horses . . xxiv 90
or cornage, in horses, cause of iii 29
Roasting, observations on . . xvii 36
Roberts, his case of injury of the brain vii 585
Robertson, Dr. J. A., on cataract iv 257
Robertson, Dr. J. H., on spinal
diseases . xii 177, 183, xiv 219
Robertson, Dr. W. H., on diet v 380, 397
on gout . . . . . xx 215
Robertson, Mr., his critique on Dr. Prout xxi 121
Robinson, Mr., on abnormal urine xviii 366
on inhalation of ether . . xxiii 547
Robiquet, M., discovered codeine ii 207
Rochoux, M., on tubercles . . i 238
Roe, Dr., on the hooping-cough . vii 210
Rœderer, and Wagler, on typhus xii 295
on examination in pregnancy . i 108
morbus mucosus . . xxiv 354
the areola in pregnancy . iv 452
Roesslin, Eucharius, on midwifery xiv 84
Rogerson, Dr., his eminence in Russia i 606
Roget, Dr., on physiology . . ix 534
on the use of plants . . iv 15
Rognetta, Dr., on ophthalmology x 2, 21,
xxi 158
on the astragalus . . xviii 124, 131
Rohuna, sulphate of . . . xvi 358
Rohun bark, account of . . xvi 358
Rokitansky, Dr., his Morbid Anatomy xv 83,
84, 105
on intestinal strangulations iii 495
Roller, on the use of the, after labour iv 398
Rollet, M., on epidemic meningitis xviii 160
Roman board of health . . . vi 576
meals, account of the . . xvi 220
mode of producing perspiration xxii 435
Romans, ancient, form of their heads xviii 448
Romberg, Dr., his clinical observations xxiv 41
on the fifth nerve . . . vii 544
nervous diseases . . xvii 407
Rome, institutions for the insane at i 498
Römer, Dr., on the arteries of the
corneal conjunctiva . . ii 235
Roods, Mr., on spinal affections . xii 177
Rooms, on ventilation of . . xix 6, 16
Roosa-ke-tel, account of . . xvi 357
Roots, Dr., on porrigo . . . iii 143
on vomiting in hysteria . . ii 200
Roots of the nerves, on the origin of v 495-98
plants, on the selecting power of iv 16
Roots of vegetables, experiments on iv 15
Rosas, Dr., on diseases of the eye v 31, 33
his Ophthalmic Report . . iii 517
Rose, Mr., his Report on Midwifery ix 220
of St. George's Hospital, notice of ii 9
Rossignol, a complaint of printers i 284
Rosy spots in typhus, remarks on . iii 260
Rot or asthma of grinders . . xxii 320
Rotatores dorsi muscles, Theile on . xiv 377
Roth, Dr., on indigo in epilepsy, &c. ii 244
Rötheln (rubeolæ) at Leith, account of x 285
Dr. Heim on . . . xiv 206
Dr. Paterson on . . . xiv 206
the diagnosis of . . . xiv 206
Rouen, account of, by Dr. Conolly xix 603
asylum, case of a lunatic in . xix 607
lunatic asylum at, account of . xix 603
Roupell, Dr., on typhus . . xii 313
on typhus fever . . .viii 428, 435
Rousseau, his apophthegm on medicine xii 22
Roussel de Vauzeme, M., on the skin ii 429
Routine, or mechanical practice . xxii 121
practice, observations on . xxi 264
practitioner, remarks on the . iv 286
Routinier practitioners, account of . xiv 403
Routinism, strictures on . . xxii 188
Roux, Prof., his celebrity in cataract vi 51
his mode of operating in cataract vi 56
operations for cataract, re-
marks on . . . x 38
treatment of abscesses . xi 218
on lacerated perineum . . xxiii 283
Rowe, Dr., his books advertisements xviii 229
on diseases of women . . xviii 229
nervous diseases . . xviii 229
specimens of his English . xviii 230
Rowland, Dr., on neuralgia . viii 529
Royal Academy of Medicine, Memoirs of xxi 408
Sciences in Prussia ii 578
College of Surgeons in London xii 576
Jennerian Institution, death of vi 491
medical appointments, English v 626
Scotch v 626
honours, remarks on . v 548
Medico-Chirurgical Society, re-
marks on the . . . ii 158
Society, prize medals of the . iii 291
proceedings of . . ii 213
Transactions of the . ii 212
suture for hernia in Turkey . vi 345
touch for scrofula, Wiseman on xxii 126, 151
and witchcraft, remarks on xix 470
Royer-Collard, M., on temperaments xviii 153
Royle, Dr., his Materia Medica . xxiii 244
his Botany of Himalayan Mountains i 215
Himalayan Botany . . iii 205
on Hindoo medicine . . vi 506
Rtischeff, the Howard of Russia i 602
Rubbing-sound between pleuræ, case of iv 298
when heard in chest . i 482
Rubefacients, various, for ascites . ii 230
Rubeola and scarlatina, diagnosis of xv 382
Rubeolæ (Rötheln), Dr. Heim on . xiv 206

Rubi Epistolæ Edinburgenses . ii 303

Rubus, Epistles of, by Dr. Fletcher ii 303

Rucelleri, the Abbé, history of . v 406

Rudius, his supposed discoveries . x 238

Rufz, Dr., on phthisis at Martinique xviii 158

Rule, examples of living by . . v 410

Rumball, Mr., on insanity . . xvi 81, 105

Rumination, probable object of . xiv 307

Rumsey, Dr., on irritative fever . viii 277

Running, pain in left side in, cause of xxiii 143

Rupia, and its varieties . . . xv 386

Rupture, cause of strangulation in . xxii 51

of the perineum, consequences of vi 536

diaphragm . . . xxiv 172

uterus, case of . . vi 539

frequency of, Dr. Collins's cases of ii 88

mismanaged cases of ii 88, 89

or vagina, in 16,434 labours . . ii 73

spontaneous . xx 531

on reduction, in mass of . xxii 201

Ruptures, Mr. Lawrence on v 547, xiv 91, 110

of perineum, treatment of . xxi 323

two general forms of . . viii 561

various treatises on . . xiv 91

Rural gormandizing, strictures on . ii 379

Russia, course of medical study in iv 270

foreign medical men in . . iv 271

history of medicine in . . i 597

medical rank in . . . iv 264

medical profession in . . iv 263

plague of, in 1551, account of i 598

in 1656, account of . i 601

scurvy in . . . . . xx 147

state of medicine in . . iv 263

statistics of longevity in . . x 582

Russian apothecaries . . . iv 263

apothecaries' shops . . iv 264

aversion to fresh air . . iv 273

barber-surgeon, his duties . iv 265

body-physician to the Czar . iv 264

candidates of medicine . . iv 271

court physicians . . . iv 267

hospital mates, discipline of . iv 264

hospitals . . . . . iv 272

diet in . . . . iv 274

law of medical prescriptions . iv 265

medical degrees . . . iv 270

examinations . . iv 270, 278

fees . . . . . iv 265

journals . . . . iv 271

literature in 1770 . . i 606

practicants . . . iv 271

salaries . . . . . iv 267

schools . . . . . iv 268

travelling fellows . . iv 271

military pharmacopœia . . xiii 54, 84

physicians and surgeons . . iv 263

poison-book . . . . iv 265

sweating, and rolling in snow . xxii 435

Rust's Medical Magazine . . i 545

Rut, in animals, escape of ova in . xix 90, 95

Rut, of the roe, observations on . xix 588

or heat, in animals . . xvii 271

Rutlandshire and Manchester, deaths in xv 305

Rutherford, Dr., his treatment of stone . . . . . xii 399

Rutter, Dr., obituary of . . ix 295

Rutty, Dr. John, account of . . xviii 37

on the diseases of Dublin . xviii 189

Ruysch on petrifying the tissues . ix 246

Ryan, Dr., a contradiction of . vi 83

his book on marriage . . v 442

censured . . v 442

obscene information . . v 444-46

Obstetric Plates . . ix 540

Obstetrician's Vade-mecum ii 523

translation of Edwards's Formulary ii 210

modesty and self-denial of . v 446

on abortion . . . . vi 81

low estimate of . . vi 86

prostitution in London . vii 539

Rye, nutritive degree of . . xvii 19

-bread, danger of ergot in . xviii 556

Ryland, Mr., his Birmingham reports ix 222

on the larynx and trachea v 424, 428

Saccharated carbonate of iron . xiii 73
Saccharine assimilation, pathology of xi 338
mal-assimilation, causes of . xi 339
principle in the stomach . . xi 332
of aliment . . . xvii 8
Sachero, Professor, his clinical report ix 223
Sac, hernial, Mr. Teale on . . xxii 194
in hernia, on not opening . xxii 377
on opening the . . vi 68
Sadler, Dr., on the use of moxa . iv 217
Safety-lamp, Davy's, observations on xv 82
Sago, experiment on feeding calves with . . . . . xxiii 499
Sailors, curious cases of wounded iv 133, 135
their freedom from calculus . vii 141
St. Antoine, l'Hôpital, account of . i 281
St. Bartholomew's Museum, catalogue of xxiii 249
St. Catherine's well, analysis of . ix 247
St. George's Hospital, report of . iii 552
St. Helena, climate of . . . xi 375
description of . . . xi 375
diseases at . . . . xi 376
salubrity of . . . . xi 376
St. Hilaire, Geoffroy, on monstrosities . . . . viii 1, 36
saying of . . . . v 48
speculations and saying of . v 339
his ideas on development . i 391
St. John Long, his liniment iii 264, vi 500
sale of his secret . . . xxii 89
some notices of . . . vi 500
St. John's Hospital, in Denmark . iii 568
St. Lambert, his definition of man v 83
St. Lucia, description of a swamp in iv 161
St. Louis, l'Hôpital, account of . i 281
St. Martin, Alexis, case of, by Dr. Beaumont . . . ii 375

St. Martin, case of, its exaggerated importance . . . ii 376
history and case of. . vi 173, 581
St. Petersburgh asylum, report of . vii 1, 51
children's hospital in . . xxi 470
endemic scurvy in . . . . i 260
foundling hospital of . . xxi 466
hospitals in . . . . . iv 277
medical academy of . iv 268, 272
essays of . . . xxi 460
mortality of . . . . . x 582
St. Saveur, spa at . . . . xiv 353
St. Thomas's Hospital Reports i 543, ii 198, iii 142
Mr. South's i 203, 208
St. Vitus's dance, causes and treatment of . . . . . iv 505
Dr. Stiebel on . . iv 505
St. Yon asylum, austere discipline in xix 606
Rouen, lunatic asylum of . xix 603
Salaam convulsion, case of . . xx 263
Salacity, morbid, in hemiplegia . viii 579
Salamander, black, development of xix 588
movements in vesicles in . xxii 264
Salamanders, experiments on . . v 514
Salicine, ague treated by . . i 571
as a substitute for quinine . i 570
cases of ague treated by . . i 571
in tic douloureux, case of . i 572
unmerited reputation of . . v 64
unsuccessful in ague . . ii 178
use of, in ague . . . xxi 212
Saline aperient waters . . xiv 325, 342
constituents of animal fluids . xxi 543
injections in cholera, effects of iii 112
formula for iii 112
medicines, in yellow fever . xxiii 428
mixture, for purpura . . iii 263
principle of aliment . . xvii 12
solutions, rapid absorption of . v 344
treatment, of fever, success of iv 535
on Dr. Stevens's . . iii 263
Salines, administration of, Dr. Bird on xx 70
Saliva, acid, and alkaline . . v 268
in gastritis, &c. . . iii 542
acidity of the . . . v 138
action and uses of . . . vii 236
of, in digestion . . xviii 529
alkaline, or neutral, in health iii 542
analysis of . . . . vii 236
chemical reaction of . . v 268
composition of healthy . . xxiii 130
concentrated, in stomach . xvi 221
contains ammonia . . . i 552
derivation of the term . . xxiii 129
digestive power of . . . xxiii 392
experiments on xvi 222
properties of . . . xxiii 134
does not coagulate by heat . i 552
Dr. Wright on . . . xxiii 129
Enderlin's analysis of . . xix 268
experiments on, by Dr. Schultz i 551
with, on dogs . . xxiii 130
great normal secretion of xvi 221, 223
Saliva, important uses of . . xiv 307
its alkaline properties . . i 552
Liebig on the use of . . xiv 512
M. l'Heritier on . . . xvii 434
mode of obtaining it pure . xxiii 129
of the horse, experiments on . i 552
original researches on . . xvi 146
pathological varieties of . . xxiii 135
physiological uses of . . xiv 239
production of tubercle from . xx 123
properties of . . . xxi 560, 563
reaction of . . . . vii 236
sulpho-cyanogen in . . xiii 260
the main agent in digestion . xvi 222
Salivary glands, extirpation of, results of xiv 221
secretion, metastasis of . . xvii 258
report on . . . xvii 258
Salivation, for 16 weeks, benefit of a v 44
in syphilis, by Dr. Bene . . i 18
mercurial, in dysentery . . xxiii 151
in syphilis . . . v 22, 23
treatment of . . . xvii 125
use of iodine in . . iii 511
Salmon, fins of smoked, diarrhea from xvii 25
putrid pickled, death from . xvii 25
Salpêtrière, la, account of . . i 281
baths in . . . . xix 290
Dr. Conolly's account of . xix 281
epileptics and idiots in . . xix 287
recitation, &c., in . . . xix 282
religious instruction in . . xix 291
separate rooms in . . . xix 286
Salt, common, effect of, on calomel xviii 545
Salt marshes, on malaria from . xiii 350
Salted meat, Liebig on . . . xxiv 272
Salter, Mr., on carditis . . ix 433
on hypertrophy of the heart . vi 387
Salts, action of, in alimentary canal xi 442
crystals of, in sputa . . xvii 521
in juice of flesh, blood, and lymph xxiv 269
metallic, tonic action of . . xi 442
neutral, effects of, on the blood xiv 144
formation of . . . xi 131
purgative, mode of action of . xi 442
which dissolve fibrin . . xx 75
Salvagnoli on the Maremma of Tuscany xxi 205
Samson-Himmelstiern, Dr., on scurvy . . . . . xx 147
Sampson, Mr., on criminal jurisprudence xvi 81, 95
on tracheotomy in intoxication iv 140
Sanative conversion of disease, cases of xxiii 591
Sanitary commission, reports of . xviii 492
condition of Dublin . . xxi 1, 14
depôts in India . . . i 345
habits, effect of civilization on xviii 244
question, Mr. Neison on the . xxi 17
questions, one-eyedness in . xxi 356
regulations, evils from want of xxi 351
M. Dupuytren on . . xvi 289
Sanden, Dr., life and works . . x 597, 598
obituary of . . . . . x 597
Sanguification, on the process of . xxi 482

Sanguineous temperament . . xv 424
Dr. Lévy on . xix 53
SANKEY, Dr., his medical instructions to seamen . . . xxiv 212
SANSON, M., on cataract . . vi 231
on excision of conjunctiva . v 38
Sap, descent of, in plants . . iv 26
in vegetables, motion of iv 20, vii 180
of plants, chemical changes in iv 22
propagation of, to leaves . iv 20
*Saper-fare* or *savoir-faire* of physicians xxii 117
Saphena on the thigh, meddling with xx 47
Saponification in dead bodies . ii 398, 400
Sappers and miners, disease of . xiii 532
Sarcocele, use of stramonium in . i 578
Sarcoma, and cancer, Dr. Lebert on xxii 31
medullary, structure of . . iii 383
Sarconotic entozoa, Dr. Lanza on . xxiii 58
Sarcosine, properties of . . . xxiv 267
SARLANDIÈRE, M., on military hygiene xix 377, 381
Sarsaparilla, its use in syphilis . v 20
observation on . . . . xxii 170
Saturnine collyria, mischief from . xviii 407
diathesis, marks of . . . x 427
SAUCEROTTE, M., on pathological anatomy . . . . . vi 293, 307
SAUNDERS, John Cunningham, tribute to ii 342
SAUNDERS, Mr., on the teeth . v 205
Sausages, on poisoning by decayed xi 444
SAUTIER, M., his method with fractures vi 319
SAVA, Dr., on the duties of the physician xxii 115
Savage life, on the diseases of . xviii 238
Savage of Aveyron, failure in educating xxiv 21
Savant, anecdote of a . . . . iv 4
Savin, treatment of rheumatism by xxi 372
Saw, on a new cranial . . . xiv 555
Sawing the bone, position of a limb in vi 167
SAWKINS, Mr., on hernia . . viii 596
SAXTORPH, Dr., on prolapsus of the cord xv 314
Scabies, cured by simple inunction xxi 530
ferina, infectious to man . xiii 49
or mange . . . . xiii 49
M. Rayer on . . . . ii 150
on the insect in . . . . xv 397
Scabious constitution, remarks on . xx 314
Scalded glottis, case of . iii 562, vi 270
Scald-head, lotions and ointments for i 578
treatment of . . . . i 578
(see Porrigo.)
Scalds and burns, M. Vidal on . xi 311
Scalenus muscle, the pleural . xix 104
Scalp, bloody tumour of . . vii 74
bloody tumours of, in infants . i 182
caution on caustics to . . xxii 172
chronic eczema of . . . . xv 116
counter-irritation of . . vii 64
impetigo of . . . . xv 117
incision of, in cerebral disease vi 381
incisions of . . . . . xxi 273
long issue in, Dr. Wallis on . xvii 96
Mr. Erichsen on diseases of xv 114, 123
skull, and brain, slices of, cured xv 177
Scalp, sloughing of back of, in typhoid fever ii 37
the hairy, wants papillary structure ii 437
Scammonii mistura, for children . xiii 82
Scandinavian Society at Gottenburg x 549
Scarborough, mineral waters of . xiv 345
Scarf-skin, thickening of, Dr. A. Combe on i 329
Scapulæ, disengaged, curious case of xvii 99
Scarification in Turkey . . iv 395
Scarifications, daily, of infants' gums xxiii 197
in quinsy . . . . . vii 550
Scarlatina, acetic acid in . . xxi 497
aconite as a preventive of . xvii 223
affections of the eye in . . xvii 221
a kind of narcotism in . . ii 178
albuminous urine in . . xvi 243
analysis of urine in . . xiii 537
anasarca after . . . xvii 221
and fits after . . ix 276
and measles, treatment of . xv 382
rubeola, diagnosis of . xv 382
smallpox together . . iv 219
belladonna as a preventive of iv 392, xvii 223
bleeding in . . . . . v 364
carbonate of ammonia in . . xvi 512, xvii 216, 224
cerebral disease after . . iii 309
change of eruption in . . xxi 471
colchicum in . . . . v 281
contagion of . . . . . xxi 365
different forms of . . . xvi 242
diseased follicles in . . ii 46
Dr. Berndt on . . . . ii 178
Dr. Chapman on . . . . xxi 364
Dr. Kennedy's treatment of . xvii 221
Dr. Williams on . . . v 363
dropsy after . . . v 363, xi 240
succeeding . . . xxiii 306
history of . . . . . xvii 216
homœopathic treatment of xvii 222, xxii 581
in Dublin in 1831 . . . xvii 217
Newcastle, Dr. Charlton on xxiv 286
puerperal women . . xix 237
its relation to the kidneys . x 319
kidneys in dropsy of . . v 364
malignant, form of . . xvii 218
in Dresden . . . xiv 204
morbid anatomy of . . xvi 338
appearances in . . xvii 217
prevalent in Turkey . . iv 392
prognosis from pulse in . . xvii 221
prophylactics against xv 383, xvii 222
report on . . xvii 562, xx 560
sequelæ of treatment of . . xxiv 45
symptoms of, Dr. Kennedy on xvii 219
the eruption in . . . xvii 220
treatises on . . . . xvii 216
treatment of . . v 365, xxi 365
ulcerations of larynx in . . xvii 218
urinary sediment in . . xxiii 504
urine in . . . . . xvi 337
wry-neck from . . . xvii 219
Scarlatinal dropsy, cause of . . xxiii 424
Scarlet fever, bleeding in . . iii 108

Scarlet fever, dropsy following . iii 262
Dr. Graves on . . xvi 241
Dr. Mackintosh on . . iii 108
Dr. Nieuwenhuys on . iii 161
Scepticism, medical, remarks on . xxiii 469
of medical men, remarks on . xxi 444
SCARPA, his error on fungous testicle i 470
SCHARLING, Dr., on vesical calculi xv 537
SCHEDEL, Dr., on diseases of the skin xii 98
on hydropathy . . . xxii 428
Scheele's green, on poisoning by . xviii 551
Scheldt, expedition to the, in 1809 . v 290
SCHELLING, his philosophy . . v 84
SCHINDLER, Dr., on traumatic ophthalmia ii 267
Schlangenbad, mineral waters of . xiv 351
SCHLEGEL, Dr., on suicide . . v 368
SCHLEIDEN on the structure of plants ix 497
SCHMID, Dr., his homœopathic practice xxii 569
SCHMIDT, Dr., his aphorisms . xix 557
SCHNEIDER, his hæmatomania . vi 515
SCHNITZER, Dr., on legal responsibility xiv 529
Scholeschiasis, disease from insects xii 219
SCHÖNLEIN, his medical system . iii 451
his natural system of diseases . xxi 25
his pathology and therapeutics xxi 20
SCHÖNLEIN'S investigations, by Remak xxiii 503
School genius, future failure of the ii 387
of observation, fault of the . vii 353
some great men dunces at . ii 387
Schools, for the sons of medical men xix 613
infant, remarks on . . i 342
medical, introductory lectures at i 203
medico-chirurgical, in Prussia i 298
military and naval medical . xvii 246
Russian medical . . . iv 268
midwifery, the first . i 604
Schriftsteller Lexicon, Dr. Callisen's xvii 504
SCHUBERT, Dr., on the endermic method xiv 530
SCHULTZ, Dr., his experiments on saliva i 551
on digestion by the cæcum . v 402
general pathology . . xx 339
the physiology of vomiting . ii 537
renewal of human life . xvi 208
spinal cord . xvii 379, 403
SCHUMER, M., on diseases of cartilage x 330
SCHWABE, Dr., on pustula maligna vii 550
Schwalbach, the water at . . xiv 355
SCHWANN, Dr., on artificial digestion iv 201
on evolution of the chick . x 229
periodic phenomena xviii 165, 178
the structure of animals . ix 495
SCHWEICH, Dr., on influenza . vii 95
SCHWEIG, M. on periodic occurrences
xviii 165, 174
SCHWENINGER, on hydrocephalus . xii 229
SCHWÖRER, Dr., on infanticide . iv 87
Sciatica, and disease of the brain . xxiv 155
cured by blisters round the leg i 499
turpentine enemata . i 569
diagnosis of . . . . . xiii 156
distinctive symptoms of . . xxiv 154
Dr. Hunt on . . . . xviii 66
Sciatica, Dr. Hunt's treatment of . xviii 66
Dr. Seymour on . . . xxiv 154
femoro-popliteal neuralgia . xiii 154
neuralgia, diagnosis of . . xvii 411
treatment of . . . xvii 411
painful points in . . . xiii 155
treatment of . . . xx 265, xxiv 154
use of ranunculus in . . xiii 232
with pain alone . . . xviii 67
with piles and prolapsus ani . xviii 67
Sciatic nerve, acute inflammation of xviii 66
chronic inflammation of . xviii 66
Science, and art, disquisition on . vi 103
and practice, feud between . xvii 472
definition of . . . . xx 141
general, its value . . . ix 428
in general, Bacon's opinion on xxiii 106
Natural Cyclopædia of . . xii 227
physical sources of . . xvii 517
poor spirit of the trader in . ix 428
Sciences, demand aid of each other i 369
dependent on calculation . xii 4, 5
non-medical, advantages of . xii 11
of life, remarks on the . . vi 103
saying of Lord Bacon on the . vi 105
Scientia scientiarum of Bacon . ix 424
Scientific acquirements of medical men iii 365
and practical medical men ix 429
Scirrhoma, Dr. Carswell on . . i 128
Scirrhous, and fungous testicle, diagnosis of . . . . i 474
cancer, Dr. Hannover on . xxiv 376
liver, state of peritoneum in . iii 76
tumour of the brain . . xx 229
tumours of breast, excision of . xxii 168
uterus, on labour with . . xi 502
Scirrhus, anatomy and varieties of . xxi 424
characteristics of . . . xi 160
composition of . . . xvii 446
of the breast, case of . . v 419
on operating on . v 416
of the œsophagus, cases of . xxiv 46
stomach, latency of . xviii 210
uterus, leeches in . . vi 95
origin of . . xviii 523
Scissors, tonsil, *plate of* . . i 268
Sclerotic, anatomy of the . . xi 69
Sclerotica, dark hue of, as a sign of death iv 198
inflammation of the . . xx 276
on thickening of the . . xvii 235
Sclerogenous deposit in plants . ix 503
Sclero-iritis, Mr. Tyrrell on . . xi 74
Scleroticonyxis, improved method of xii 548
Sclerotitis, diagnosis of . . ii 345
Scolding servants, salubrious . i 357
Scoliosis, causes of . . . v 137, 142
effected in sleep . . . xix 370
lateral spinal curvature . . v 136
muscles concerned in . . v 137
Scolopendra, heart and arteries of . xx 498
Scorbutic formations, remarks on . xx 150
Scorbutus, condition of the blood in xvii 150

Scorbutus, pectoral effusions in . xii 250
Scorpion, circulating apparatus of . xx 500
Scotch, Dr. Pirondi's opinion of the viii 534
Scotland, and Scotsmen, remarks on xxi 166
legal relief to the poor in . xi 27
management of the poor in . xi 1
mortality of troops in . . vi 150
peculiar epidemic fever in . xx 233
state of female labourers in . xi 26
wretched state of the poor in . xi 26
Scott, Dr., on the education of idiots xxiv 1
Scott, Mr., his plastering of joints v 462
on cataract . . . . . xvii 329
inconsistencies of . . xvii 330
Scott, Sir Walter, Thomas Carlyle on xviii 481
writing in the morning . . i 342
Screaming in children . . vi 426
Scribblers, crawling medical . . xxi 441
Scrofula, and phthisis, connexion of xviii 486
contrasted . . xix 22
deaths from . . xxii 135
identity of . xxii 132, 137
independence of . xix 22
and tubercle, identity of . ii 597
causes of . . . . . xxii 141-5
climate in relation to . . xviii 490
diseased cartilage in . . xiii 169
Dr. Bene on . . . i 19
Dr. Glover's prize essay on . xxiii 513
Dr. Tyler Smith on . . xviii 231
effect of civilization on . . xviii 247
efficacy of cod-liver oil in . xiv 445
endemic . . . . . xviii 490
essential nature of . . . xxiii 518
external causes of . . . xviii 490
fatal, without external sign . ii 18
from deficient ventilation . xviii 500
nurse's milk . . . xviii 489
general existence of, in families xviii 484
hereditariness of . . . xxii 142
remarks on . . . xxiii 519
hereditary origin of . . xviii 482
humidity in relation to . . xviii 490
Indian climate injurious in . i 346
in children, report on . . xvii 565
in factories . . . . xxii 148
influence of climate on . . xxii 147
seasons on . . xxii 154
in lambs, cases of . . xxiv 88
manufacturing districts . xv 312
prisons, causes of . . xxiv 138
iodine and barium in . . xxii 152
lactation in relation to . . xxii 145
may be induced in any habit . ii 19
mineral waters useful in . xiv 342
M. Lugol on the causes of . xviii 481
Mr. Phillips's treatise on xxii 124, 155
Mr. Toynbee on causes of . xviii 500
mud baths and poultices in . xiv 342
nature and character of . . xxiii 514
non-contagious . . . xviii 491
on its passing a generation . xviii 488
ordinary curability of . . xviii 231
Scrofula, particular remedies in . xxiii 520
pathological causes of . . xviii 489
pathology of . . . iii 446
predisposition to . . . xxii 142
prevalence of, in different countries xxii 138
prevalent among brutes . . xxiii 518
question as to the nature of xxii 127, 129
relation of, to gout . . viii 242
relative prevalence of . . vi 19
report on . . xx 564, xxiii 308
root of the elder in . . iii 508
royal touch for . . xxii 126, 151
sea-water and bathing in . xxii 153
Sir A. Carlisle on . . . vi 188
state of the blood in . . xxiii 517
statistics of . . . . xxii 125
success of cod-liver oil in . xxi 467
syphilis as a cause of . . xviii 487
table-salt in relation to . . xviii 232
three diseases of lungs from . xvi 252
treatment of xiv 342, xxii 150, xxiii 519
use of cod-liver oil in . . viii 554
ray-liver oil in . . viii 554
walnut-leaves in . . xii 539
value of chalybeates in . . xi 209
Scrofulosis and tuberculosis . . xiii 339
Scrofulous, and tuberculous diseases xxii 29
constitution, remarks on . xx 314
signs of . xviii 482, xxii 128
deposit, characters of . . xxii 130
diathesis, complexion in . xxiii 517
diseases, cod-oil in . . ix 553
in animals . . . xxiv 88
operations in . . . ii 13
families, mortality in . . xviii 485
habit, characteristics of . . viii 193
matter, derivation of . . xxii 131
ophthalmia, affection of nose in xxiii 373
conium in . . . viii 256
parents, acquired health of . xviii 487
original health of . . xviii 486
products, chemistry of . . xxiii 515
subjects, effect of spring on . xviii 491
swellings, iodine in . . viii 522
tubercle, Pott's disease from . i 575
Scrotal calculus, Prof. Chelius on . ii 260
Scroti pruritus, cured by lemon-juice xii 548
Scrotum, acute anasarca of . . ix 443
anatomy of the . . . xvii 55
invagination of, for varicocele . xi 529
numerous lymphatics in . ii 443
operation for restoring . . vii 412
plastic operations in . . xxi 312
ulceration of, rare in fungous testicle i 472
watery swellings of . . v 468
Scudamore, Sir C., his formula for
inhalation . . . . v 606
his letter on gout . . . vii 534
his visit to Graeffenberg . . xxii 428
on inhalation . . . v 606
Scurvy, affections of lungs and spleen in xx 152
cause of . . . . xx 148
effect of civilization on . . xviii 253

Scurvy, effusions caused by . . xx 149

endemic, in St. Petersburg . i 260

epidemic, report on . . xx 271

from bad nutriment . . xiii 492

inflammatory diathesis in . i 263

in United States' army . . xvi 44

marine, treatise on . . xx 147

peculiar state of the gums in . xx 151

production of . . . xiii 492

sea, use of nitre in . . viii 595

Scybala in typhoid fever, M. Chomel on ii 40

Sea anemone, anatomy of the . v 211

Sea, and air, connexion of temperature of ii 522

and earth, on temperature of . xxii 229

air of, preservative from cold . xiii 390

where most salubrious . xvi 501

benefit of sending the sick to . xxi 378

life, sanitary effects of, on lungs xv 15

on coasts, temperature of . ii 520

-service, effect of civilization on xviii 243

-sickness, etiology of . . xv 69

remark of Müller on . xv 69

remarks on . . . v 152

soonest on long waves . xv 68

-water in scrofula, Mr. Phillips on xxii 153

Sealy, Dr. H., his Medical Essays iv 196

strictures on his essays . . iv 196

Seamen, benefit of change of station to xi 184

Drs. Sankey and M'Arthur on xxiv 212

health of, in West Indies . xi 184

on the use of spirits by . . xxiv 532-5

Searle, Dr., on the why and the wherefore . . . . xxiii 579

Seasonal mortality, tables of . . xviii 202

in three cities . . xxiv 95

Seasoning, or acclimatization . xxi 378

Season, influence of, on health xxiv 92, 113

on phthisis . . xxiv 105

Seasons, chemistry of the four . xxiii 238

healthiness of, in England . xi 432

influence of, table of . . xviii 172

marriages in different . . xviii 202

mortality in, in relation to age . xxiv 113

wet, less mortality in . xxiv 102, 113

Sebaceous follicle of eyelids . . xviii 420

glands, and hair-follicles . ii 453

of the skin . . xv 380, xxi 200

Sebadilline, Prof. Turnbull on . ii 499

Sebastian, M., his Elements of Physiology . . . xxi 282

on gout and scrofula . . viii 242

labial glands . . . xv 227

pocks in smallpox . . viii 247

tubercles . . . iv 216, vii 553

Secale cornutum, dangerous use of . iii 164

different strength of . iv 99

Dr. Hamilton on . . iv 402

essay on . . . . xii 509

experiments on . . x 554

observations on . . xiii 70

successful use of . . v 577

Secernent gland-cells, development of xix 565

glands, structure of . . xiv 291

Seclusion of the insane, Dr. Conolly on xi 546

Secondary diseased actions from injuries &c. . . . ii 9

inflammation, Mr. Travers on . ii 13

symptoms, proportions of . ix 237

Second editions, on pseudo- . . xvii 531

sight, Scottish belief in . . xix 470

Secreted fluids, on corpuscles in . xiv 293

Secreting organs, on stimuli of the . xvii 479

Secretion, and absorption, physiology of xiv 564

nutrition, connexion of xiv 293

secreting structures xx 293

by the red corpuscles of blood xiv 597

defective, on remedies for . xvii 479

diseases of, backward . . xvii . 479

Dr. Williams on . . xvii 478

forward . . . . xvii 478

Dr. Carpenter on . . . xv 279

examples of abnormal . . xv 280

force of, Dr. Clark on . . i 391

laws of primary organ of . xiv 565

Mr. Addison's doctrine of . xviii 96

Mr. Goodsir on . . . xiv 564

nervous agency in . . . vii 179

system in . . . xiii 477

peculiar in a gouty person . xiv 227

performed by cells . . . xv 279

probable efficient agents of . ix 523

true, and fluid exhalation . xiv 564

Secretions, analysis of . . . viii 346

anomalies of . . . . xiii 338

disordered, case of . . . vii 125

elective attraction of organs for xiv 145

Haller's opinion on . . vii 283

nutritious, of plants . . iv 26

special, in plants . . . iv 26

Sects, medical, of Germany . . xvi 472

Sedative influence of hydropathy . xxii 453

Sedatives, and stimulants, remarks on v 203

in insanity, Dr. Conolly on . xxiv 152

Dr. Steward on . . xxii 60

observations on . . . xvi 191

three modes of exhibiting . xviii 67

Sedentary life, diseases from . . xx 103

Sédillot, M., his self-complacency xiii 368

on empyema . . . . xiii 364

Sediments, urinary . . . vii 162

Seeds, almost indefinite vitality of . vii 174

dispersion of . . . . iii 367

latent life of . . . . v 556

long latent vitality of some . iv 130

oily, nutritious properties of . xvii 19

Ségalas, M., on urethroplasty . xxi 408

Segato, Sig., his petrified works . ix 246

on animal petrifaction . . ii 532

Seguin, on pulmonary exhalation i 242

on the treatment and education of idiots . . . . . xxiv 1

Segur Dupeyron, M., his case of plague . . . . xxiv 258

Seidlitz, mineral water of . . xiv 345

Seidlitz on hemorrhagic pericarditis i 259

Seidschütz, mineral water of . . xiv 345

Selections in Children's Diseases . ii 353

Diseases of Women . xiv 531

Self-abuse, observations on . xv 360, 362

preventives of. . xv 360, 362

Self-consciousness, and suffering . xxi 105

on morbid . . . xviii 479

Self-control, in insanity . . xvii 245

observations on . . xxiv 238

Self-instruction of medical students xxii 161

Self-limited diseases, Dr. Bigelow on iii 200

Selwyn, Dr., on bronchocele . vii 586

Semeiology, Dr. Watson on . . xvii 123

in diagnosis . . . . xx 309

M. Piorry on . . . . vi 128

Semen, and ovum, meeting of . xvii 272

appearances of . . . xv 352

detection of, on linen . . xvii 443

human, Dr. Davy on . . vi 551

nature of action of, on the ovum xvi 522

microscopic diagnosis of . . xv 352

Semicircular canals of ear, functions of xvi 369-72

Seminal corpuscles in river fish . xiii 233

emissions, diurnal . . . xv 351

morbid, cure of . . vi 532

nocturnal . . xv 351, 355

entozoa, remarks on . . viii 141

involuntary discharges . . xv 347

vesicles, Dr. Faye on . xv 346, 361

weakness, causes of . xv 348, 366

cauterization in . . xv 355

moral symptoms of . . xv 354

symptoms of . . . xv 353

treatment of . . xv 355, 360

Semitic nations, distribution of . xxiv 456

Seneca, quotation from . . vii 500

Senega, its effect on the heart . i 264

M. Lombard on the virtues of iii 510

mode of administering . . iii 510

Senegal, topography and diseases of xi 364

Senile gangrene, remark on . . xxii 172

hectic fever, remedies in . xvii 108

inflammation, description of . xvii 107

Sennæ syrupus, formula for . . xiii 82

Sennertus on plagues from earthquakes . . . . xiii 497

Sensational conceptions, Unzer on . xxiv 190

instincts, Unzer on . . . xxiv 199

Sensation, absence of, in insects . xxi 494

absent without the brain . xxiv 204

and consciousness, Cullen on . v 528

its seat, Dr. Arnold on . xvi 177

perception distinct . . v 503

cerebrum not the seat of . xxii 504

common, modifications of . v 111

Dr. Carpenter on . . . v 531

Dr. Johnstone on . . . xi 505

excited action without . . v 512, 516

in animals . . . . viii 510

law of irradiation of . . xvii 409

laws of . . . . . xvii 408

loss of, in fifth nerve . . vii 544

meaning of the word . . xvi 172

mode of . . . . xxiv 132

Sensation, muscular action without. iii 38

on the share of nerves in . vi 217

production of . . . ix 102

remarks on the term . . v 502

sympathy in relation to . . iii 34-9

transference of, remarks on . xi 499

Sensations and impressions, remarks on v 503, 537

change of the, in drunkards . ii 479

external, and sensibility . . xxiv 189

irradiation of . . . . xvii 393

morbid, in the eye . . . xx 285-87

transference of . . . iv 443

Sense, case of a girl with only one . x 288

on delusions of . . . xx 15

organs of, and viscera, history of xx 419

Senses, mesmeric transposition of xix 448

special, organs of, report on xix 584

the lost—deafness, Dr. Kitto on xxi 188

Sensibilities, blunted, in medical men xxi 442

Sensibility, and selfishness, female . v 408

confined to the brain . . v 523

in brain, nature of . . . v 523

in lead colic, lesions of . . v 60

of different individuals . . viii 377

remarks on the term . . v 502

the characteristic of animals . iv 6

Sensitive plant, on the motions of . v 488

young ladies, remarks on . v 408

Sensorial fibres, voluntary change in xii 67

functions in asphyxia . . xii 279

torpor, Mr. Newnham on . xv 421

Sensorium, the cerebrum not the . xxii 512

Sensori-volitional nervous fibres . xvi 159

Sensory columns, decussation of . xxiv 576

common, Prochaska on . . xxiii 205

ganglia and cerebrum, on . xxii 504

functional distinctness of xxiii 235

Sentient impressions . . . xxiv 188

Sepoys, rate of mortality of, in Egypt vi 152

Sepulture, intra-mural, evils of . xv 542

laws against . . xv 516

Sequence, law of, in natural phenomena xxi 528

Sequestra in tubercular disease of the spine . . . . i 576

on absorption of . . . xv 30

Seraphim Hospital in Stockholm . xxii 459

Serapion, midwifery of . . xiv 83

Sero-cystic tumours of female breasts xxii 167

of the breast . . x 587

Serny, Dr., on spinal curvature xii 177-78

Serous apoplexy, remarks on the term iii 312

cavities, accidental, close . xviii 81

cysts, iodine injections in . xiii 543

dropsy, Professor Vogel on . xxii 325

effusion, Dr. Gross on . . xxiii 66

effusions, mostly alkaline . xvii 442

inflammation, observations on xxii 386

membranes, adhesion of . . vi 232

Dr. Hodgkin on . . iv 31

gangrene of . . . iv 52

inflammatory products of xxiv 119

osseous deposits in . . iv 53

Serous membranes, report on . xix 256
structure of . . . xxii 385
tubercles in . . iv 53, 54, 245
ulceration of . . . iv 51
ultimate structure of . iv 32
Serous tissue, miliary tubercles in . iv 54
tissues, inflammation of . . iv 34
nerves in . . . iv 33
secretions of . . . iv 34
vascularity of . . . iv 33
Serpents, mechanism of movements of xv 204
poison of . . . . viii 315
venomous, on the bite of . xix 367
on the fangs of . . xx 401
Serratula arvensis, noxious to oats iv 130
Serratus magnus, case of paralysis of xvii 99
energizer of the . . v 139
function of the . . v 139
Serre, Prof., on deformities of the face xix 396
Serres, M., his idea on monstrosity xii 385
his theory of development . viii 10
Sérullas, his reduction of arsenic xiii 184
Serum and albumen ovi, mixture of viii 350
milky . . . . . . xix 255
in diseases . . . xx 74
salts of, in Bright's disease . xvii 151
Setten, Dr. von, on saliva . . vii 236
Seton, in disease of the brain . . xiv 252
in ununited fracture . . xii 255
Setons, Dr. Hall's surprising success
with . . . . . . xxiii 198
in encysted tumours . . ix 441
on thread . . . . . ix 578
on the use of, in paraplegia . xiii 261
Seven half-crowns swallowed, case of iv 251
Seven, operation of the number . xviii 170
Severn, Vale of, fevers in the . vi 9
phthisis frequent in vi 22
topography of . . vi 4
Sewalik Hills, fossil zoology of . xxi 476
Sewell, Dr., double amputations by vi 550
Sewerage and drainage of towns . xxi 339
Sewers in towns, enormous defects in xxi 338
public, of the Greeks and Romans v 450
Sewer-water, as manure, value of . xxi 357
Sex, of the child, in relation to parturition xix 541
of the embryo, Dr. Carus on . i 101
prognostics of, before birth . i 101
Sexes, on the peculiarities of the . xii 65
Sextus V, Pope, anecdote of . xxiii 162
Sexual attachments and beauty . vii 373
coition, effect of, on the lungs ix 324
nervous influences . . . xii 65
organs, diseases of, in animals xxiv 87
propensity, observations on . xxii 513
on the seat of . . . xxii 534
Seymour and Conolly, Drs., compared . . . . . xxiv 156
Seymour, Dr., friendly advice to xxiv 155
his thoughts on diseases . . xxiv 139
his treatise on dropsy . . iii 429
on dropsy, estimate of . . iii 439
Shaking hands, organ of . . xix 482
Shampooing and friction in sprains iv 232
Shark, great white, large jaw-bones of xx 392
Sharks, and rays, cerebellum in . xxii 536
fossil, prodigious . . . xx 392
Shapter, Dr., his case of loss of
memory . . . . iv 478
on Exeter . . . . vi 382
intermittent fever . . xii 93
plague . . . . . xii 92
South Devon . . xiv 470, 477
the mortality of Exeter . xx 523
yellow fever . . . xii 94
Sharkey, Dr., on digitalis in epilepsy xi 509
Sharling, M., on calculi . . ix 360
Sharp, Mr., on injuries of the head xiii 123
Sharpey, Dr., his Quain's Anatomy xxi 499
on ciliary motions in animals . i 509
on the formation of bone . xxiv 386
Shattuck, Dr., his translation of Louis x 494
Shaw, Mr. A., on facial palsy . v 281
on Sir C. Bell's discoveries . ix 96, 121
the effects of rickets . . xviii 371
the mode of physiological
researches . . . xiv 568
Shaw, Mr. J., in relation to Sir C. Bell ix 140
Shearman, Dr., on animal and
vegetable life . . . xxi 473
Sheath, in tenotomy, not divided xiii 2
Sheep, ancon, or otter breed of . vii 376
curious breed of, in N. America xxiv 62
effect of climate on . . xxiv 61
temperature of . . . xii 143
Sheffield, statistics of . . . xvii 528
the grinding of tools at . . xviii 303
Shells, classification of . . . xxi 81
membranous substance of . xxi 79
prismatic cellular substance in xxi 77
structure of . . . xx 376, xxi 62
Sheppard, Mr. E., his translation of
Gibert . . . . xx 521
Sheppard, Mr. J., on insanity . xvii 526
strictures on xvii 526
tribute to xxiii 219
Sherwood, Mr., death of . . iv 568
Shingles, M. Rayer's pathological
remark on . . . ii 149
mode of relieving pain in . vii 530
sometimes intensely painful . ii 149
Ship, a medical, suggestion of . xv 15
Ships, merchant, list of medicines for xxiv 213
of war. (See Navy.)
ventilation of . . . xix 20
by pipes . . . xviii 222
Shock after injuries, treatment in . xix 529
effects of, on nervous system . xiv 57
Shoes, on measuring for . . xii 179
Shoolbred's powder for spleen disease xiii 363
Shops, on medical men keeping . x 219
Short- or near-sightedness, lenses for xvi 362
Short-sightedness, means of curing x 567
M. Guerin on . . . xii 554
myotomy for . . . xii 253
Shot in bottles, on poisoning by . xviii 546
Shoulder, dislocation of, myotomy in xiii 21
old dislocation of, fatal reduction of xiv 41

Shoulder or arm presentations in 16,434 labours . . . ii 72
Shoulder-joint, dislocations of . v 569
excision of . . . iii 482
Shoulder presentations, danger of perineal laceration in . ii 75
Dr. Collins on bleeding in ii 79
Shriek, in prussic-acid poisoning . xxiii 423
Shumer, Dr., on cartilages . . vii 535
Siamese twin monster . . . xii 377
twins . . . . . . viii 34
account of . . . ii 299
unseparable . . . xii 378
Sibilant rhonchus in bronchitis . i 487
sound in the smaller bronchi . i 482
Sibson, Mr., on the site of organs . xix 103
Sichel, Dr., disingenuousness of . xvi 114
his contradictions . . . xvi 117
on glaucoma . . . . . xvi 110
Sick, duties of medical men to the . xxi 448
Irish poor, shocking picture of ii 184
room, life in the . . . xviii 472
management of . . xi 511
Sickness, annual, table of . . xxi 16
Side, pain in left, in females . . x 44
Siebergundi, Dr., his case of viper bite i 582
Siebert, Dr., on medical diagnosis xx 308
Siebold, Dr., his History of Midwifery xiv 80
on abortion . . . . . vi 81
on ciliary motions . . . iii 502
Sierra Leone, insalubrity of . . xi 367
medical topography of . xi 365
temperature of . . xi 367
Sigaultian section in midwifery . xii 489
Sight, defective, lenses proper for . xvi 362
in the deaf, observations on . xxi 194
Mr. W. W. Cooper on . . xxiii 510
Sigmoid valves, rupture, &c., of . xvi 325
treatment of spinal curvature . xii 186
Sigmond, Dr., on animal magnetism v 598
on mercury . . . . . x 245
mesmerism . . . vii 349
tea . . . . . . . ix 241
Signoret, M., his detection of arsenic xiii 192
Signs, and symptoms, distinction of xvii 123
remarks on . . xvii 499
from displacement of viscera . iv 292
motions of the thorax . iv 292
shape and size of thorax . iv 293
the sense of touch . . iv 292
insufficiency of alone . . iv 291
of disease, general and physical viii 467
on due combination of . . iv 297
on one side only, value of . iv 296
physical, and symptoms . . iv 291
Dr. Gerhard on . . iii 186
Dr. Williams on . . xvii 499
in reference to time . iv 297
referable to acoustics . . iv 293
restricted meaning of the term iv 289
Singultus, or hiccup, observations on xxiv 173
treatment of . . . . . xxiv 174
Silesian society at Breslau . . ii 581
Silex in plants, remarks on . . iv 9
Silica, solubility of, in water . . xi 144
Silk factories, unhealthiness of . xx 103
ligatures for arteries . . xxii 92
manufacture, on health in . xv 299
Silkworm, muscardine of . . ix 400
Silver, chloride of, on the use of . xii 567
nitrate of, remedial action of viii 361
utility of . . . xiii 61
oxide of, in menorrhagia . xxiii 292
its therapeutical value . xx 203
teaspoon swallowed, case of . v 252
Silvester, Dr., on phlebitis . . xiv 53
Simia satyrus, on the skull of . x 251
Simiæ, term of life of the . . iii 373
Simon, Dr., on animal chemistry . xx 206
on medical deontology . xxi 438
the physiology of nutrition viii 549
the thymus gland xx 159, xxi 566
Simplicity in practice, remarks on xxiii 469
Simpson, Dr., on ether in midwifery xxiii 547, 568
on fœtal malformation . . viii 594
peritonitis . . . vii 284
hemorrhage post partum . xxiii 284
leprosy . . . . xiv 244
the sex of the child . . xix 541
Sims, Dr. John, obituary of . . vi 594
on diseases of the brain . iii 1, iii 301
on softening of the brain . iii 313
Sinapisms, proper temperature of . i 570
Sincipital combustion, use of . . vii 63
Singing in relation to hemoptysis . xxi 366
Singultus arthriticus, Sauvages on . vi 373
Sitz-bath in hydropathy . . xxii 439
Siva blamed for gastroperiodynia . iii 216
Size, great variation of, in spleen . i 565
Skate, sympathetic nerves of the . iii 490
Skey, Mr., on spinal curvature . xii 177, 181
on the treatment of ulcer . v 195
venereal disease . . xi 508
Skeleton, bone not necessary to the xxiii 476
Mr. Elkington on the . . iii 205
on the muscles of the . . v 340
report on the . . . xix 571
the, a double ossific cell . . xxiv 135
Skeletons, endo- or neuro-, of animals xxiii 474
exo- or dermo-, of animals . xxiii 474
of fishes, study of . . . xxiii 479
mankind, on varieties in . xxiv 71
Sketch, biographical, of Dr. Ley . iii 586
Skilled or habitual actions . . xxiv 193
Skin, the absorption by, Dr. A. Combe on . . . . . i 332
Dr. Madden on . . vi 332
anatomy of the . . . vi 333
and physiology of the . xv 378
and alimentary canal similar . v 344
its appendages . . xix 566
animalcule of the . . . xxi 200
blood-vessels of the, M. Gurlt on ii 436, 450
case of remarkable torpor of . iv 220
circulation in the . . . ii 436, 450
coloration of, in infants . . xi 199
calorific apparatus of the . ii 445, 451

Skin, the colours of the, theory of . ii 452

congenital deficiency of, case of xx 549

disease of skin . . xvii 549

diseases of . . . . . xx 266

arrangement of . . vi 427

baths in . . . . . i 497

and fumigations in . iv 180

diseases of, Cazenave on . . xxi 491

causes of ignorance of . xv 114

chronic, treatment of . xiv 341

classification of x 549, xv 373, xxi 492

cured by smallpox . . xxiii 308

constitutional nature of . iv 179

depletory measures in . iv 179

Dr. Schedel on . . xii 98

Dr. Thomson on . . x 250

empirical practice in . iv 175

on French practice in . iv 176

from constipation . . x 45

heat in . . . . . xix 184

in animals . . . xxiv 85

on local depletion in . vi 428

mercury and antimony in iv 179

Mr. Dendy on . . vi 425

Mr. E. Wilson on . . xxiii 251

Mr. Hunt on . . . xxiv 280

Mr. Plumbe's divisions of . iv 176

M. Rayer on . . . ii 147

nature of . . . vi 427

obscurity of . . . iv 175

on exhibiting arsenic in . xxiv 281

on *offhand* practice in . iv 176

remedies in . . . iv 180

severity of, in France . iv 176

sulphuret of lime in . iii 242

sulphur fumes in . . iv 180

sulphurous waters in . xiv 341

treatises on . . . . xv 372

Dr. Combe on the mucous coat of the i 330

effluvia absorbed by . . i 331

electric properties of . . xx 312

excessive tenderness of . . vi 143

exhalation from the . . i 330

experiments on functions of . xx 106

function and pathology of . xxii 450

functions of, Dr. A. Combe on i 330

general structure of . . xxiv 130

healthy, Mr. E. Wilson's treatise on xxi 198

hemlock baths in diseases of . vi 538

hereditary hypertrophy of . ii 459, 568

relaxation of . . . ii 568

infantile gangrene of . . vii 474

inflammatory products of . xxiv 122

inhalant apparatus of . . ii 440

its non-contractile property . iv 170

layers of . . . . . xv 379

mammillary nervous apparatus of ii 432

medication by . . . v 343

miasmata absorbed by . . i 331

M. Gibert on diseases of . xx 521

morbid anatomy of, in smallpox v 469

mucific apparatus of . . ii 445

M. Gurlt on . . . ii 449

Skin, nervous apparatus of . . ii 434

nitrate of silver in diseases of . v 287

of forehead, black secretion from xxiii 416

mankind, on the colour of . xxiv 68

on it becoming mucous membrane xii 428

papillæ of, described . . ii 432

functions of . . . ii 435

peculiarities of wounds of . x 351

plates of, explanation of . . ii 615

Plumbe and Green on diseases of iv 175

rheumatism of . . . xiii 234

scaly diseases of . . . xv 394

sebaceous glands of . . xxi 200

short hairs of, remarks on . xxi 202

seat of colour of . . . vii 239

stony concretions in . . xiii 240

structure of the . ii 429, vii 239, x 349

Plates I and II . . ii 431

sudorific apparatus of . . ii 437

the older anatomists on . . ii 429

transplantation of . . . vii 394

varnishing of, asphyxia from . xix 568

venereal diseases of . . x 381, 420

ventilation of, necessity of . xx 109

Skin-bound disease of infants, causes of ii 364

dissection of ii 362

pathology of ii 363

treatment of ii 364

Skoda, Dr., Disquisition on Acoustics by . . . . . xiv 174

his erroneous acoustics . . xiv 173

notion on bronchophony . xiv 173

treatment of pneumonia . xxii 591

on percussion, &c. . . . xiv 173

Skrimshire, Dr., his Medical Guide vii 513, 516

Skull, and brain, slices of, cured . xv 177

aperture of, protection of . xiii 135

depressed fracture of, treatment of xv 176

effects of rickets on the . . xviii 371

filling up of openings in the . xii 226

fractured in parturition . . xi 253

medico-legal case of . ix 62

fracture of bones of, in labour xvii 549

of the, during labour . iv 233

of, flux from ear in . . xv 174

of inner table of . xv 174, xv 401

through the orbit . xv 178

fractures of, by contre-coup . xv 173

the base of the . . xv 173

human, on the form of the . xxiv 381

its thickness in lunatics . . v 373

loss of part of, case of . . xv 400

on the national form of . . xviii 372

puerperal hyperostosis of . xix 392

punctured fracture of . . xiii 133

starlike fracture of . . . xiii 133

structure of, in early life . xvii 371

unprotected aperture of, case of xiii 135

Skulls, American, comparative view of x 474

fallacy respecting . . . xxiv 383

figures of six different . . ix 211

form of, Dr. Zenne's scheme of xxiv 385

European and Negro compared xviii 386

European, Negro, and Chinese, table of xviii 385

national, comparison of . . ix 211

of animals, remarks on . . xvii 372

human races, diversities of . xxiv 72

the ancient Peruvians . . x 478

oval or elliptical . . . xxiv 73

prognathous and pyramidal . xxiv 72

Slack, Mr., his paper on primary tissues iv 6

Slade, Dr., on ophthalmia . vii 217, 220

remarks on his style . . vii 217

Slate, discovery on the nature of . iii 582

Slaughter-houses in towns, remarks on xxi 340

Slave-prisons in Norway, account of xvii 88

Slavonian race, remarks on the . xviii 379

Slavonians, form of their skulls . xviii 375

Sleep, amount of, necessary to man xiv 502

anatomy of, Dr. Binns on . xv 184, 186

a promoter of hemorrhage . x 63

condition of the brain in . v 386

Dr. M. Hall on the cause of . xxiii 194

how to produce it at will . xv 187

magnetic or mesmeric . . xix 432

remarkable case of . . v 599

mesmeric, operations during . xxii 477

nervous, Mr. Braid on . . xix 428

of infants, amount of . . xiv 502

the respiratory ganglia . xix 306

on promoting respiration in . vi 584

spontaneous . . . xix 440, 443

when dangerous in arachnitis . xxii 407

Sleeping-tea for children . . xxi 355

Sleeping with clothes on and off . xix 16

Sleepiness, apoplectic, Dr. Hull on . xvii 201

Sleeplessness from anemia, remedy for xxii 54

in old age, remedies for . . xvii 110

Sleep-waking, phenomena of . . xx 16

Sloet, Baron, his cure for epilepsy iii 263

Sloths, fossil, dental structure of . xxi 73

Sloughing, in dysentery . xxiv 339, 343

in typhoid fever . . . ii 37

use of iodine in . . . viii 521

Smallpox after vaccination . . vi 493

Smallpox, anatomy of epidermis in v 470

the cutis in . v 471

and cowpox, on affinity of vi 482, 485, 492

on deviations in . . vi 491

identity of vii 204, vii 295, ix 76, 79, xiv 51

non-identity of . . xiii 560

and scarlatina together . . iv 219

vaccination, paper on . xiv 50

arising without contagion . i 370

Avicenna's account of . . vi 483

before the Christian era . . vi 483

bleeding in . . . . iii 109

Boerhaave's idea on . . vi 483

causes of late prevalence of . ix 77

deformity prevented in . . xi 246

destruction of eye after . . xvii 234

Dr. Chapman on . . . xxi 358

Dr. Mackintosh on . . iii 109

eetrotic methods in . . xv 384

effect of civilization on . . xviii 254

Smallpox, exclusion of light in xiv 553, xv 383

first recorded case of . . vi 483

frequency of secondary . . vii 208

great mortality from . . vi 495

human, infection of cows by . xiv 397

identity of with cowpox . viii 614

in a child before birth . . xiv 235

animals, Dr. Baron on . vi 482

Layard and Viborg on . vi 482, 483

Calcutta, report on . . xx 349

Denmark, Dr. Wednt on . v 207

England, 1837-9 . . xi 432

London, calculations on . xi 433

the camel . . . . x 281

the cow, inoculation from . x 472

Wirtemberg . . . vii 186

inoculation of . . . xii 97

evils of . . . . vi 484

history of . . . vi 484

of Catherine II, for . i 605

in India . . . xx 354, 356

in relation to cicatrices . . xiv 51

insect origin of . . . xiv 553

its identity with cowpox . i 376

known in ancient Asia . . xxiii 542

local use of mercury in . . vii 552

matter, experiments with . ii 252

mercurial inunction in . . viii 421

plasters in . . . ix 249

plaster in . . . xi 246

morbid anatomy of . . x 469

skin in . v 469

mortality from . . . . ix 353

in Calcutta from . . xx 350

of formerly . . . vi 486

Mr. Eagle on . . . vii 278

nature of the pocks in . . viii 247

on cutting short eruption in . xv 384

puncturing pustules in . vi 563

origin and history of . . xxi 358

prevention of marks by . . xxi 361

pitting in . . xv 383

pustule, anatomy of . . xxiii 307

in the bladder . . x 287

pustules on the conjunctiva . x 32

renewed generation of . . xxi 359

report on . . . . . xvii 562

salutary effects of . . . ii 250

same origin of as cowpox . vi 482

second attacks of . . . vi 483, 492

singular susceptibility of . vi 492

skin-diseases cured by . . xxiii 308

sometimes incommunicable . xxiii 318

statistics of . . . . . x 588

summary of morbid anatomy of v 479

tables of mortality of . . xii 96

the cutaneous glands in . . v 472

fila sudorifera in . . v 472

genital organs in . . v 478

hair in . . . . v 472

larynx and trachea in . v 476

mouth and tongue in . . v 473

nasal cavities in . . v 476

Smallpox, œsophagus in . . v 475

pharynx in . . . v 475

serous membranes in . v 478

stomach and intestines in . v 476

treatment of . . . . . xxi 360

unsusceptibility of some to . xxi 360

use of opium in . . . i 313

SMEE, Mr., his Elements of Electro-metallurgy . . . xv 541

his new splints . . . vii 583

on electro-metallurgy . . xi 510

physical science . . xvii 517

the potato plant . . xxiii 581

Smegma preputii, secretion of . xix 567

Smell, and taste, nerves of . . xviii 143

organs of, Dr. Heron on their diseases . . . . . i 270

*versus* reasoning faculties . xi 16

S., Mr., wounded in a duel, case of . xxiv 326

SMITH, Dr. Ash., on yellow fever in Texas xii 433

SMITH, Dr. D., on fever in Glasgow xviii 179

SMITH, Dr. F. W., on diseases of cæcum i 501

SMITH, Dr. H. H., on the spinal cord v 224

SMITH, Dr. N. R., on palate suture i 265

SMITH, Dr. S., his Philosophy of Health . . . i 313, v 380

on putrid effluvia . . . xi 9-13

SMITH, Dr. Tyler, advice to . . xviii 232

on scrofula . . . . xviii 231

strictures on . . . xviii 231

SMITH, Dr. W. G., on tetanus in Hayti v 229

SMITH, Mr. John, on the food of man xxi 148

SMITH, Mr. H. L., on the origin of disease, &c. . . . . xvi 510

SMITH, Sir Francis, on creosote . iv 251

Smoke in towns, observations on . xxi 342

SMYTH, Dr., his medical contributions xix 237

Snail, development of shell of . xxi 82

preparation of a . . . xv 210

Snake, a living, in the stomach . viii 257

Snakes, effect of prussic acid on . xii 138

poison of, Dr. Davy on . . xii 137

poisoning by, treatment in . xii 138

Sneezing, remarks on the nature of vii 175

severe, cured by snuff, case of . ii 245

spasmodic, emetics for . . xvii 420

SNOW, Dr., on the inhalation of ether xxiii 573

Snuff-chewing by American ladies . v 412

Snuff, &c., on mixing various kinds of xvi 199

plaster, benefit from . . viii 582

severe sneezing cured by . ii 245

Soap consumed in London, value of xviii 496

Societies, medical, in Prussia . . ii 577

remarks on . . ii 577

secret Pythagorean . . xxiii 91

friendly, observations on . xxi 16

medical, caution to . . iii 376

English . . . . . viii 535

scientific in Norway . . iv 548

Society for natural and medical science at Berlin . . . . . ii 582

medical, of Bombay, account of xxi 374

of Medical Observation . . xix 390

Medico-chirurgical, new Royal ii 158

Sod bi-borate, use of, in stone . xii 398

Soda, carbonate of, bad effects of viii 342, 344

medicinal effects of . viii 344

Mr. Neville on . . iii 190

Sodium, as an element of food . xvii 4

chloride of, oxide of, in fever . v 563

use of to the blood . . xxiv 270

SOEMMERING, his Anatomy of the Viscera . . . . . xx 419

his museum . . . . . xiv 374

new edition of his Anatomy . xix 373

Sœurs de la Charité, as nurses . i 494

not very gentle . . . i 494

Softening, central, of spinal cord . v 182

cerebral, M. Pinel on . . xx 35

transient palsy in . . xxii 406

two kinds of . . . xvii 529

Dr. Carswell on . . . i 137

of arterial coats . . . i 158

brain, acute . . . xvi 1

curability of . . . iii 313

diagnosis of . . . vi 144

Dr. Cross on . . . xxiii 72

Fuchs on . . . xi 464

in children . . . xxi 390

memoir on . . . xiii 535

on red . . . . iii 312

remarks on . . . iii 312

results of . . . x 260

rigidity of limbs in . . iii 313

supposed cure of . . xxii 462

traces of cure of . . iii 313

treatise on . . . xvi 1

yellow . . . . iii 312

heart, diagnosis of . . viii 464

Dr. Latham on . . xxiii 181

mucous membrane of intestines in children . . ii 551

spinal cord, nature of . . iv 144

stomach, cupri sulphas in . i 257

Dr. Dürr on . . . i 257

Soil, effects on, by excretions of plants iv 27

in relation to cholera . . xxiii 337

remarks of Dr. Lindley on . iv 16

source of nitrogen in . . xvii 225

Solanæ, on the extracts of the . iii. 229

Solar lamp, account of the . . xix 18

Soldier, cost to government of each xxii 184

Soldiers, amusements of . . xix 386

and officers, mortality of . viii 222

clothing and dress of . xix 384, xxi 173

diet of . . . . . . xix 385

duty and exercise of . . xix 386

education of . . . . . xix 387

in India, causes of diseases in . ix 564

on discharging and pensioning xviii 118

enlisting, &c. . . viii 537, xviii 117

preserving the health of . xix 377-89

punishments of . . . xix 388

sick, new litter for . . . xxii 225

Soles of feet, tickling, in paralysis . v 519

Solidism, on the doctrines of . . vi 296

Solitary confinement, bad effects from xv 56

effects of . . . iv 191

Solitary glands, lesion of in dysentery xxiv 339-43
of the colon . . . xxiv 335
intestines . . i 527
Solly, Mr., his work on the brain . iv 485
on mollities ossium . . xx 100
the brain, commendation of iv 488
glands . . . ix 533
human brain . . xxiv 572
optic nerve . . . v 593
Sol-lunar influence on diseases . xxiii 588
Solon, Dr. Martin, on albuminuria, or dropsy . . vii 121, x 301, 328
Somervail, Dr., on tobacco . viii 582
Sommer, Dr., on the signs of death iv 197
Somnambulic ghosts . . . vii 342
phenomena . . . . vii 332, 336
Somnambulising, immorality of . vii 334
Somnambulism, a materialist converted by . . . . iv 447
cases of . . . . xix 443-6
clairvoyance in . . . vii 334, 337
"en rapport" in . . . vii 332
ideas of weight in . . . xxiii 228
impious absurdity in . . vii 346
lucid . . . . . . xix 447
magnetic . . . iv 442, vii 331
remarks on . . . . . xix 307
responsibility in . . . . x 151
spontaneous . . . xix 440, 446
state of . . . . . iii 328
system in . . . . iv 442
sympathy in . . . . . vii 336
vision in . . . . . xix 465
Somnambulist, instructive case of a . vii 326
Somnambulistic state, definition of xix 441
Somnambulists, shooting exploits of vii 329
Sond à dard in lithotomy . . xii 194
Sonde à demeure, objections to the viii 156
Sonnenstein, lunatic asylum at, good i 499
Sonorous rattle in the larger bronchi i 482
Sool, a name for gastroperiodynia . iii 215
Soot, decoction of, preparation of . v 575
in diseases of the bladder . v 575
Sopor, hystericus, hot poker for . xvii 202
Sores, malignant, use of heat in . xix 186
on cleaning from pus . . xv 46
on Lisfranc's mode of dressing xv 46
primary syphilitic, treatment of x 398
Sormani, Dr., on sudden deaths . x 420
Souberbielle, M., on lithotomy . xii 194
Souffle placentaire, auscultation of . i 90
remarks on the . . xiv 366
uterine, diagnosis by . . i 91
Soul-force, animal, of the brain . xxiv 186
Soul, a form of, in plants . . v 525
in animals, Galen on the . xv 422
seat of, nausea at the . . vii 229
the rational, of Stahl . . xxiii 585
Sound, different, of right and left lung . . . . . . iii 188
for nasal duct, figure of . . xxi 311
idea of, by those born deaf . xxi 197
modification of a vesical . ix 260
Sound, propagation of, in the ear . viii 85
uterine, used by Dr. Simpson . xxiv 508
Sounds, cardiac, their extent and direction . . . . . i 484
in the chest, observations on . xiv 176
new pulmonary . . . ix 315-19
of water moving through a tube v 590
properties of respiratory . ix 302
straight, easy passage of . xii 410
Soup, from bones in France, account of xiii 494
good and cheap, for the poor . xiii 496
Liebig's prescription for . . xxiv 272
observations on making . . xxiv 271
Soups, strong, not really nutritious . ii 377
well digested . ii 377
Sour springs, in North America . viii 359
South American coast, salubrity of xv 6
naval command . . xi 192
South America, mortality in navy in xv 6
South, Mr., his description of the bones . . . . . iii 491
his Translation of Chelius xx 110, 119
his St. Thomas's Hospital Reports i 203, 208
Southey, Dr., his studying in the morning . . . . i 342
on continuous mental work . xiii 412
Space, on the bodies of . . xix 156
Spain, medical journals of . . i 304
reform in . . . i 608
physician-surgeons of . . i 304
present state of medicine in . i 303
pure physicians of . . . i 304
Spa, mineral water at . . . . xiv 355
-physicians, on books by . xiv 318
Spas, gluttony and degradation at . ii 372
haunters and denizens of . i 334
medical writer on, portrayed . xiv 315
mineral, on the benefits from . xiv 314
of England and Germany . xiv 309
pilgrimages to . . . xiv 309
staple medical advice at . . i 333
Sparman, his story of prolapsus uteri . . . . . . vi 237
Sparrows, general notions shown by xi 93
Spasm, and stricture, belladonna in ii 261
from pinching the dura mater . xiv 58
languor, &c., Dr. Wilson on . xvi 418
of the face, cured by myotomy xiii 22
glottis, Dr. Joy on, referred to ii 240
heart, remarks on . ii 338
treatment of . . xxiii 184
muscles of neck, cases of iii 263
respiratory muscles . xvii 420
stomach, agreeable advice on v 391
ground biscuit in . xxiii 267
Spasms of the face, myotomy in . xiii 244
the, or cæliac neuralgia . . xvii 416
Spasmodic affections, sulphur in . x 586
treatment of . . v 275
contraction of uterus . . viii 51
diseases, electricity in . . vi 72
from pericarditis . . ix 431
movements in pericarditis . xxiv 557

Spasmodic tic, Dr. Hall on . . xiii 158
Spatula for displaying the throat . xxiv 502
Spawn, fish, long latent vitality of . iv 130
Specialization, the principle of . xiii 167
Special pleading in medical writings xviii 492
Species, and genus, on the terms . iii 371
observations on the word . xix 176
on the origin of . . . . xix 180
Specific disease, remarks on the term vi 352
gravity of parts of the body . xii 134
inflammations, pathology of . iii 18
medicine, new system of xxii 565, 567
Spectacle-vendors, medical names to x 40
Spectacles, black-crape, objectionable xvi 363
convex, in asthenic amaurosis . xv 246
Mr. Curtis's wire-gauze . . v 404
on different kinds of . . xvi 362
Spectral illusions, case of . . iii 261
observations on . xxiv 132
Speculum, penchant for, in Paris . xii 367
revolt in Paris against the . xii 367
Speculum auris, invented by Hildanus iii 87
of Dr. Kramer and others iii 88
polish within unfit . iii 88
uteri, apparatus for . . viii 530
Dr. Ashwell's . . . vi 71
Dr. Churchill on . . vi 91
made of glass . . v 255
Mr. Fergusson's new . xxiii 292
Mr. Jones on . . . viii 529
on the use of . . viii 530, xxi 175
vaginæ, Dr. Balbirnie on . iii 175
of Paulus Æginetus . xiv 209
on the use of . . . ii 514
Spèdale di Bonifacio at Florence . i 498
Spedalskhed, or Norwegian leprosy xviii 346
Spedikati, his work on Scurvy, notice of i 606
Speech, and language, remarks on . vii 228
by the deaf, observations on . xxi 190
loss of, in typhus, relief of . v 138
Spencer, Earl, on gestation of cows xi 273
Spender, Mr., on mortification . iv 259
Spermatocele, new operation for . vii 444
Sir A. Cooper on . . . vii 444
Sperm, experiment on, by Dr. Harlan ii 520
Spermatic cord, hydrocele of . . xvii 73
varicocele of . . vii 444
discharges, M. Lallemand on . xv 346
Spermatorrhœa, predisposing cause of xxiii 508
treatment of . . . . vi 532
Spermatozoa, chemical characters of xiv 572
description of human . . xv 353
figures of human . . . xv 353
in hydrocele, cause of . . xx 84
in ordinary hydrocele . . xviii 371
observations on . . . xvi 522
of the camelidæ . . . xvi 287
on cysts containing . . xx 100
rarity of, in the ovary . xvi 520, 556
real character of . . . xv 265
remarks on . . . . viii 141
report on . . . . xvii 270
Sphincter ani, new pin for lacerated iv 469
Sphincter ani, spasm of . . xvii 420
Sphincter in imperforate anus . xviii 455
Sphincters, nature of their action . v 510
Sphinx ligustri, nervous system of iii 291, v 620
Sphygometer, account of Mr. King's iv 365
Spider, house, dissection, and *plate of* i 538
Spiess, Dr., on Van Helmont . xvi 403
Spikenard, oil of, Dr. Forsyth on . xvi 357
Spillan, Dr., curious observations of xiv 141
his Jurisprudence of Insanity . x 129
Manual of Practice . . vi 202
Medical Formulæ . . vi 202
Pharmacopœia . . . iv 101, 103
Therapeutics . . . xiv 140
translation of Andral . i 188
on materia medica, &c. . . ix 541
Spina bifida, new operation for . xii 546
on the cure of . . xiv 233
radical cure of . . xii 546
remarks on . . . viii 22
successful treatment of x 102, xxiii 300
Spinal abscess, case of . . . iii 222
accessory nerve, functions of . v 310
nerves, functions of . xi 517
affections, Dr. Laycock on . xii 60
and cardiac affections, connexion of v 126
heart-disease, relation of . xii 126
nervous diseases . . xiv 219
bodies, nervous . xviii 134, 136, 139
canal, continuation of . . xviii 135
vascularity in, deceptive . v 122
circulation, peculiarities of . xii 126
column, cancer of, cases of . xiv 53
congestion of . . xii 126
complaints, on exercise in . viii 500
cord, affected in Beriberi . v 119
affections of . . . iii 31
a ganglionic centre . . xvi 160
anatomy of . . . v 496
and brain, action of . v 523, 593
connexion of xviii 426, 438
diseases of . . xxi 381
injuries of . . iii 10
and its nerves, connexion of xxi 459
anterior column of . . xiv 222
blood-vessels of . . xvii 388
case of disease of . . xi 451
cases of lesions of . . iv 378
circulation of . . . xiv 379
collection of serum in . vii 248
commissures of . xvii 389, 391
concussion of . . . iv 144
connexion of fibrils of . xvii 390
consciousness in . . v 534, 537
diseases in relation to . iii 32
diseases of . . . ix 493
Dr. Budge on . . . xvii 400-3
Dr. Nasse on . . . iv 415
Dr. Smith on . . . v 224
effect of gently withdrawing iii 37
elementary anatomy of . xvii 386
experiments on iv 416, 418, v 224
fasciculi of, in brain xviii 429, 438

Spinal cord, fibres of, arrangement of xvii 401

fibrils of . . . xvii 389

fluid in, and uses of . xiv 469

functions of iv 415, xiii 481, xiv 238

and action of v 523, 537, 593

Dr. Stilling on . xvii 400

columns of . . xii 245

halves of . . xii 235

regions of . . iv 419

Van Deen on . . xvii 395

gelatinous substance of . xvii 388

general physiology of . xxii 275

gouty affection of . . ix 493

gray matter of . . xvi 183

substance of . . xvii 387

history of its physiology . v 523

horizontal fibrils of xviii 135, 142

independent power of . xvi 180

inflammation of, cases of xv 403

influence of, on the bladder iv 424

on brain . . . iv 420

genitals . . iv 425

intestines . . iv 423

nutrition . . iv 426

secretions . . iv 426

the heart . . iv 423

uterus . . iv 425

vitality . . iv 426

injuries of . . . iv 143

in relation to sympathy . iii 34-9

intimate structure of . xvii 388

Legallois' opinions on the v 530

longitudinal fibrils of . xvii 392

mental relations of . . iv 416

morbid affections of . iii 29

M. Fouville's new views of xviii 424, 425, 427

Mr. Grainger on . . v 486

his doctrine on . v 499

Mr. Mayo on . . . v 534

Mr. Solly's anatomy of . iv 486

new anatomy of . . xviii 425

of the frog . . . vii 542

pathology of . . iii 29, ix 437

peculiar action of . . xvi 160

physiology of . . v 486, xvii 391

Dr. Arnold on . . xvi 178

psychical influence of . x 258

reflex function of . . iii 33

relation of, to respiration . iv 421

nerve-roots to . . xxii 277

report on . . . xvii 265

rheumatism of . . xiv 412

sensibility of . . . v 515, 534

softening of . . . x 52

after injuries . . iv 144

Stilling's experiments on xvii 395

structures of . . . xix 577

sympathetic affection of . xii 67

symptoms of disease of . iii 30

termination of, a ligament iv 486

the true, its extent . . xvi 159

transverse fibrils of . . xvii 392

Spinal cord, treatment of lesions of iv 379

treatises on . . . xvii 379

Van Deen's experiments on xvii 393-9

vital unity of . . . iv 419

white substance of . . xvii 387

curvature, apparatus for . . xii 185

cause of . . . . x 364

causes of . . . v 384

dissection of . . . xiii 564

Dr. Harrison on . . xii 178

Dr. M. Hall's treatment of xxiii 188

Dr. Stromeyer on . . v 133

dry-cupping in . . xii 183

early diagnosis of . . v 144

effected in sleep . . xix 370

extension in, absurd . x 365

hop-scotch in . . xii 180

incision in . . . ix 554

lateral . . . . v 136

causes of . . v 137, 142

treatment of . . xviii 226

M. Malgaigne on . . x 364

Mr. Childs on . . xiii 221

Mr. Stafford's views on . xviii 227

myotomy in . . xii 283, xiii 564

on growing out of . . xii 181

operation for . . . xiii 566

prevention of . . . xii 188

rate of amendment in . xii 181

recumbence in xii 178, 180, 188

seat of, in the loins . xii 182

section of muscles in . xii 182

state of muscles in . . iv 409

table of cases of . . xii 180

treatises on . . . xii 177

treatment of . . iv 512, v 142, xii 177, 179, 180

deformity, equilibrium exercises in xvi 347

gymnastics in . . xvi 346

lever-belt for . . xvi 347, 350

mechanism for . . xvi 346

Mr. Harrison on . . xvi 341

disease, case of . . . vii 446

convulsions from . xii 123

from gout . . . xvi 256

inferences from cases of . iii 30

Mr. Key on . . . vii 446

on pain in . . . xii 124

serious errors in . . xviii 63

use of iodine in . . viii 523

ganglion, structure of a . . xxiv 130

hysteria, bad practice in . . iv 136

hysterical affections . . iv 136

incurvation, mechanical remedies for v 141, 143

inflammation, fatal case of . xxii 460

injuries, conditions after . . iv 143

gangrene in . . . iv 146

muscular spasms in . iv 145

paralysis after . . iv 144

priapism in . . . iv 145

reaction in . . . iv 146

respiration in . . iv 145, 146

Spinal injuries, sentient nerves in . iv 145

symptoms in . . . iv 146

temperature in . . iv 146

treatment of . . . iv 147

urinary organs in . . iv 145

irritation, a dubious affection . i 460

anomalous cases from . xii 105

case of . . . . xxi 99

caused by congestion . xii 128

causes of . . . xii 106

Dr. Bennet on . . xii 105

Dr. Griffin on . . iv 252

Dr. Huss on . . . xxii 463

Dr. Marshall on . . i 459

his cases of . . i 463-7

on defective . i 468

Dr. Saunders on . . i 459

Dr. Türck on . . . xix 96

from leucorrhœa . . xii 106

instructive case of . . xii 125

its indistinctness . . i 460

observations on . iv 252, xxi 101

only neuralgia . . xiii 152

pain on pressure in . . xxii 464

phenomena of . . xix 97

state of cord in . . xii 105

symptomatic . . . xix 96

table of cases of . . iv 253

tenderness in . . . xii 106

treatise on . . . xiv 533

treatment of xii 125, xvii 417, xxii 465

various authors on . . i 459-60

lateral curvature of, cure of . xii 177

machine for . xviii 226

the face in . . v 141

lesions, seminal emissions in . iv 425

marrow, diseases of, in animals xxiv 89

Dr. Van Deen on . . xii 514

gelatinous substance in . vii 502

heat after division of . viii 197

intimate structure of . vi 400

its influence on the heart ii 132

minute structure of . vii 503

partial wound of . . xi 525

pathology of . . . xii 535

respiratory tract of . . viii 480

softening of . . . v 182

Stilling's mode of slicing xviii 140

structure of . . . vii 541

true, Dr. M. Hall on . xvii 171

urine, in diseases of . xvi 339

muscles, section of . . viii 557

nerve-roots, report on . . xix 577

nerves, Dr. Macartney on roots of ii 135

fibres of roots of . . vii 504

Mr. Shaw on . . . ix 130

origin of the posterior . iv 417

report on . . . xvii 268

roots of . . . v 496, xxiv 576

functions of . . xix 558

Sir C. Bell on . . v 521

sensory roots of . . v 521

Sir C. Bell's theory of iv 414, 417

Spinal neuralgia, Dr. Romberg on . xvii 417

liniment for . . . xvii 417

treatment of . . . xvii 417

neurosis, treatise on . . xxi 280

pain in intermittent fever . viii 67

paralysis, constipation in . iv 423

rheumatism, in dogs . . xxiv 89

softening, two kinds of . . xvii 529

system, in the vertebrata . ix 101

true, anatomy of . . xvi 167

Dr. Hall's . . xvi 167

pathology of . . xvi 169

physiology of . . xvi 168

tenderness, remarks on . . vii 529

Spine-elongator of Mr. Stafford . xviii 227

Spine, angular curvature of . . xviii 225

condition of, in old age . . i 234

curvature of, Dr. Hall's stays for xxiii 189, 191

Mr. Hare on . . . ix 244

myotomy in . . . xiii 22

curvatures of, operation for . viii 557

dislocations of . . . vi 161

distortion of, Mr. Ayre on . ii 603

division of muscles of . . xix 370

essays on diseases of . . xviii 225

fracture of, cured by extension xvii 98

importance of attention to . xii 127

inflammation in, remarks on . xii 128

lateral curvature of . xiii 221, 566, xviii 225, xxiv 578

causes of . . xviii 225

cure of . . xii 177

raising depressed part of . vi 162

right convexity of, cause of . xv 342

rupture of, by muscular effort . xiii 542

sigmoid extension and flexion of xii 186

supposed diseases of . . viii 500

syncope from tap on . . xii 106

tenderness of . . . iv 253

in ague . . . . viii 67

treatment of diseases of . . vi 161

tubercular disease of . . i 574

dissected . . i 575

usual site of tubercles in . i 575

Spines of sturgeons, Prof. Owen on xxiii 482

Spino-occipital articulation, disease of v 278

Spiral arrangement in plants . iv 10

fibres of plants . . . ix 503

vessels of plants . . . iv 7

Spirometer, on detecting disease by xxiv 327

Spiro-vesicular theory of M. Raspail iv 10, 11

Spirit-drinkers, disease in . . ii 480

-drinking, before dinner . . iv 242

by the poor, remarks on . ii 389

evil effects of . . . i 364

-ration to troops in India . xxi 375

Spirit, use of, in dissecting nerves . xi 495

Spirits, ardent, remarks on drinking ii 388

on abolition of, in the navy xxiv 532-35

Spirits and ghosts, mesmeric . . vii 342

Spiritualist school of philosophy . xxiii 99

Spitalfields, remarks on the state of i 363

Spitta, Mr., on cyanosis . . xxiv 324

Spittal, Dr., on the sounds of respiration . . . . vi 584

Splanchnic inversion . . . viii 25

Splancho-skeleton of animals . . xxiii 475

Splay- or flat-foot, cause and treatment of viii 399

cured, case of . . . viii 399

treatment of . . . ix 229

Spleen, the, abscesses of . . vii 469

account of cells in . . . xix 571

a diverticulum . . . xxiii 143

a reservoir for excess of blood i 565

Aristotle's physiology of . xv 447

blood-vessels of . . . xiv 542

changes in by typhoid fever . ii 49

chronic disease of . . . xiii 363

Celsus on . xiii 363

treatment of . xiii 363

inflammation of . . i 564

congestion of, described . . i 564

cure of engorgements of . . xi 236

deranged structure of, cases of xxiv 329

diminution of, by quinine . vi 141

diseased, Celsus on . . iii 346

hemorrhage in . . iii 345

state of the blood in . iii 345

native Indian remedies for iii 346

ulcers in relation to . iii 345

disease of . . . . . xiii 362

causes of . . . iii 345

in children . . iii 345

females . . . iii 345

iodine and iron in . xiii 363

iodide of lead in . xiii 364

its analogy to chlorosis iii 346

Shoolbred's powder for xiii 363

symptoms in , . iii 345

treatment of . . iii 346

diseases of . . . . . xx 222

calomel injurious in . xvi 566

diagnosis of . . . vii 469

Dr. Bright on . . vii 468

in India . . . . i 563

Mr. Annesley's account of xiii 363

treatment of . . . vii 469

engorgement of, causes of . xix 31

treatment of . . . xix 32

enlarged, on mercury in . . iii 347

enlargement of . . i 564, xix 74

in drunkards . . . xvi 219

examination of . . . vi 140

functions of . . . . xv 447, 455

glands of . . . . . xiv 543

good health without, for years xix 571

granulo-capillary basis of . xiv 542

healthy proportions of . . vi 141

induration of, Dr. Voigt on . i 566

intervesicular spaces of . . xiv 541

liquid of . . . . . xiv 542

lymphatic vessels of . . xiv 543

the Malpighian bodies in . xxi 567

minute structure of . . xiv 541

M. Piorry's mode of percussion of vi 141

Mr. Twining on diseases of . iii 344

Spleen, pathological changes in . vii 468

proportions of, in disease . vi 141

removal of . . . . xx 244

secondary abscess in . . iii 504

spontaneous rupture of, case of viii 248

structure and functions of . viii 593

ten anatomical elements of . xiv 541

theory on the use of . . xxi 569

vascular corpuscles of . . xiv 542

vesicles of . . . . . xiv 541

weight and dimensions of . iii 199

Splenic cachexia, description of . iii 345

juice, M. Burggraeve on . . xxiii 144

Splenalgia Bengalensis, account of . i 565

congestionis, described . . i 564

Splenemphraxis, symptoms of . i 564

Splenitis, acute, treatment of . xiii 363

a rare disease . . . iii 344

chronic, symptoms of . . i 564

Splicing the main-brace at sea . xxiv 534

Splints, use of, in chorea . . xii 284

on the use of, in fractures . vi 308, 310

Spoglio, operation of . . . xxiv 257

Sponge, as a plug in uterine hemorrhage vi 85

auscultatory experiments with . ix 302

regarded as a poison . . xviii 533

Sponges, use of, in displaced uterus xviii 26

Sponging, cold, in fever, fallacy on xxii 447

Spongiole of plants, remarks on . iv 13, 17

Spongioles of plants, action of . iv 22

Spontaneous amputation in fœtus . ix 564

combustion, remarks on . . xvi 74

human combustion, case of . iv 222

version, case of . . . iii 243

Spooner and Smart, Messrs., retrospective address of . . xv 228

Spooner, Mr. W. C., on the horse's foot xi 224

Spore, the, in mosses and ferns . vii 183

Spores of plants, remarks on the . iv 28

Spots, rose-coloured, in typhoid fever ii 36

in typhus different from petechiæ . ii 40

rosy, in typhus, remarks on . iii 260

Spotted fever, Dr. Wilson on . . iv 535

Sprain, Lisfranc on treatment of . xiv 17

Sprains, friction and shampooing in iv 232

Sprengel, his definition of observation . . . . xxiii 104

Spurzheim, Dr., on phrenology . ix 190

striking mistakes of . xxii 523, 525

Sputa, black, causes of . . . iv 150

questions on . . . iv 150

in phthisis, Dr. Gellerstedt on xxiii 435

microscopic characters of . xvii 519

various ingredients of . . xvii 519-21

viscid rusty, in pneumonia . iv 316

Squamæ, syphilitic . . . xxiii 361

horny . . . xxiii 362

Squamous diseases, M. Rayer on . ii 155

treatment of . . ii 155

eruptions in animals . . xxiv 86

Squill, endermic use of . . . v 351

Squint-clippers, leer of the victims of xviii 414

on ignorant . . . xvi 271

Squinting, cured without operation . xiii 239
cure of . . . . . . x 288
by operation . . x 585, 587, 588
eye, cause of amaurosis in . v 42
power of vision of . . xi 477
hook in operation for . . x 585, 586
operations for cure of . . ix 558
Sir C. Bell on operations for . xi 492
surnames from . . . xi 474
treatises on . . . . xi 474
Squire, Mr., on morphia . . viii 275
Sri Madusa dana Gupta, his edition of Susruta . . . xxiii 521
Staberoh, Dr., on British and Irish fever . . . . . xii 318
Stables and cow-houses in towns, on xxi 342
Stafford, Mr., his case of cut-throat xi 459
on curvature of spine . . xviii 225
dissection-wounds . . iv 140
lancetted stilettes in stricture i 534
the prostate gland . . . x 528
Stagner, Mr., his new truss for hernia iii 234
Stahl, his expectant system . . xxiii 585
his physiological opinions . i 385
the Anima of . . . . i 385
Stains, chemical, from copper . xi 54
various substances xi 43
of antimony . . xi 40
arsenic . . xi 40
like arsenical, production of . xiii 184
on linen, tests of . . . v 266
Stallard, Mr., on the treatment of fever . . . . . xxiii 269
Staminal principles of Dr. Prout . xvii 12
Stammering, cause of . . . xii 209, 210
cure of . . . . . vi 253, xx 135
dangerous operation in . . xii 252
Dieffenbach on operations for xxi 331
excision of tonsils for . . xii 213
first operation for . . . xii 257
new mode of curing . . v 568
on amputating uvula for . . xii 213
operations for . . xii 210, 211
results of operations for . . xii 554
ruthless operations for . . xii 209
Sir C. Bell on . . . xii 209
"Standing," on physicians of "some" iv 287
Stanger and Wood, Messrs., their trusses . . . . . vi 343
Staninought, case of murder by . xvi 109
Stanley, Mr., on disease of spinal cord xi 451
on luxation of hip-joint . . xiv 59
pulsating tumours of bone . xxiii 412
ruptured ureter . . . xx 83
tumours of the pelvis . . xiv 64
Stanley musk deer, blood-corpuscles of xvi 287
Stannius, Dr., on the action of strychnia . . . . . v 221
Staphyloma corneæ, Mr. Tyrrell on xi 68
iridis . . . . . . x 37
Dr. Jäger on . . . iv 482
uveæ of Mackenzie . . x 37
Staphyloma, formation of . . x 37
Staphyloma, Mr. W. Jones on . v 612
partial, reduction of . . xi 68
sclerotic, dissection of a . . iii 521
treatment of . . xvii 235
singular operation for . . x 37
Staphyloplastic, etymology of . vii 411
operation . . . . vii 411
Staphyloraphy, Dr. N. R. Smith on i 265
Starch bandage in fractures, objections to . . . . vi 315-17
Starched bandage, gangrene from . viii 558
Starch, an alimentary principle . xvii 9
as food, experiments on . . xiii 490
change of, by digestion . . iv 205
conversion of into oily matter xxiii 502
digestion of . . . . xxi 562
in food, on conversion of . xxiii 501
in fractures, substitutes for . vi 316
in plants, nature of . . ix 497
invention of for fractures . vi 314
sugar, and gum, similar . xiv 304
Stark, Dr., on dropsy after scarlatina iii 262
Starvation, alcohol, how useful in xxiv 544
death from cold in . . . xxiv 544
deaths from, in England . . ix 356
effects on the stomach by . xvii 146
experiments on . . . xvii 344
morbid effects of . . . viii 524-25
mortality from . . . xiii 493
Starved dogs, state of the blood in xvii 145
State-medicine, and general hygiene xiv 446
Encyclopædia of . . xv 225
Statice armeria, diuretic powers of iii 511
Statics and dynamics, remarks on . v 322
Statistical etiology, remarks on . ix 351
method in medicine . . xxiv 292
nomenclature, uniform . . ix 351
nosology, remarks on . . ix 351
results of amputations . . iii 237
Statistics, Danish medical . . x 583
in medicine, the abuse of . ix 460
meaning and origin of term . xii 12
medical . . . . . vi 1
and moral . . . vi 17
Dr. Casper on . . xxiv 91
principles of . . . xii 1
of diseases . . . . . v 262
fever in Belfast . . . iii 268
insanity at Charenton . i 272
in Norway . . . i 278
midwifery, report on . . xxiii 291
the London Hospital . . iii 270
use of, to medicine . . ix 346
vindication of . . . ix 344
vital, contributions to . . xxi 1
explanation of . . iii 41
of England . . . xviii 197
Stature of man, observations on . xvii 274
Stays, some of the evil effects of . v 145
and tight lacing, Mr. Coulson on iii 174
displacement of the liver by . i 234
of the lungs by . . i 234
fatal case from tight . . ii 143

Stays, for crooked spine, Dr. M. Hall's xxiii 189-91

influence of, on phthisis . . xvi 453

injurious effect of . . . . i 234

sternum protruded by the use of i 234

tight, a great cause of disease ii 144

use of, sternum protruded by . i 234

to weak persons . . v 408

Steam-baths, on the advantages of xix 374

Steam-engine compared to living body vi 303

Steam in the treatment of wounds vii 438, 442

Steatopyga of the Hottentots . xxiv 451

Steatosis of the liver, Mr. Mayo on iii 77

Steel sparks on the cornea, removal of ii 269

Steinau, Dr., on hereditary diseases xvii 247

Steinbrenner, M., on vaccination xxiii 307

Stephen's remedy for stone . . xii 391

Stercoraceous concretions, percussion in i 487

Stereoscope, description of a . . vii 280

of Mr. Wheatstone . . x 18

Sterility, Dr. M. Hall's new remedy for xxiii 193

his physiology of . . . xxiii 191

on a cause of . . . xxi 90

treatment of . . . . xxiii 192

Sternum, fissure of . . . viii 22

necrosis of . . . . . xv 399

new bones of . . . . viii 248

protruded by use of stays . i 234

Stethoscope, an auxiliary only . viii 467

best form for a . . . . v 267

Dr. Billing's remark on the . v 200

Dr. Latham on the . . ii 480

Dr. Stokes on the . . . . iv 295

great utility of the . . . . ii 481

improved by Dr. Granville . vi 581

in labour, Dr. Collins on . ii 77

its utility in labour . . iv 404

mode of learning use of . . ii 477

obstetric, Dr. Hohl's . . i 87

practice with the . . . iv 295

principles of . . . . . v 267

use of, in fracture . . . xiv 6

in midwifery . . . . v 596

use of the stopper of the iv 295, 322

with flexible tube . . xi 544

wonders worked with . . ix 343

Stethoscopic tact of M. Chailly . xiv 372

Stethoscopist, the Young . . xxii 209

Stevens, Dr. A. H., on lithotomy vii 505

Stevens, Dr. W., on the theory of respiration . . . . . ii 216

Stevens, Mr. R., classical deficiency of xiii 205

his nosology . . . . xiii 202

Stevenson, Mr., on cataract . iii 207

Steward, Dr. J. B., on dyspepsia . xxiv 579

on insanity . . . . . xxii 58

Stewart, Dr. A. P., on typhus fever xii 294, 319

Stewart, Dr. D., on smallpox in Calcutta xx 349

Stewart, Dr., J., on diseases of children xiii 222

Stewing of food, remarks on . . xvii 37

Sthenic diseases, Dr. Del Chiappa on i 250

Stieglitz, a first interview with . xxiii 544

conduct and character of . xxiii 546

letter to, and account of . xix 123

on the theory of congestion . iii 210

Stieglitz, Professor Marx's tribute to xxiii 544

Stiff joint, treatises on . . . xviii 353

joints, compression and extension in xviii 363

neck, articular species of . xi 250

treatment of . . . . vi 355

Stiffness, after asphyxia, late . . ii 425

of joints, from long rest . . xiii 243

Stilettes, lancetted in stricture . i 534

Stillborn child, restoration of . viii 596

children, remarks on . viii 52

restoration of . . viii 474

report on . . xxi 352

statistics of . . iv 234

infants, cold water to . xii 560

Stillicidium lachrymarum, Lawrence on xi 77

Stilling, Dr., his experiments . xvii 395

on the nervous system xvii 379, xviii 134

Van Deen's experiments xvii 395, 399

Stimulants, and sedatives, remarks on v 203

caution respecting . . . vii 264

changes in system by use of . ii 479

internal, remarks on . . xxii 441

observations on . . . xvi 189

Stimulating by alcoholic liquors . xxiv 545

drink, on abstinence from . ii 388

effects of hydropathy . . xxii 445

Stimuli, vital . . . . . vii 175

Stimulus, remarks on the term . iv 429

Stinks in towns, observations on . xxi 335

Stitch, during exercise, true cause of xxiv 175

in the side, remarks on . . vi 516

Stitches, nervous, remarks on . i 24

Stockholm, report of diseases at . xxii 459

Stokers, peculiar diarrhœa of . . xiii 231

Stokes, Dr., his lectures . ix 461, 494

on cancer of the lungs . . xv 430

diseases of the chest . iv 285, v 423

his education . . . iv 286

wine in typhus . . . viii 281

Stomacace in children, description of xiv 203

Stomach, acids in . . . xi 332, 343

-affections, on the tongue in xix 26

a grasshopper fed with its own ii 136

altered secretions of . . xi 413

anatomy and physiology of . xix 269

and bowels, perforation of . xviii 534

relation of, to happiness ii 380

brain, relation between . ii 370

sympathy of . . xi 506

colon, displacement of . xiv 580

œsophagus, digestion of . xvi 310

pancreas, diagnosis of diseases of . . . xviii 398

renal diseases, Dr. Prout on xvi 477, 486

arthritic affections of, in old age xvii 115

as the medical doorway . . xxii 442

cancer and ulcer of . . vii 241-42

cancer of . . . . . xxii 296

diagnosis of xviii 210, xxii 297

Dr. Barras on . . xviii 208

external . . . xxiv 274

yeast-plants in . . xxiii 510

case of a living snake in . . viii 257

ulcer of . . . vii 243

Stomach, case of visible action of . ii 376

changes of, in typhoid fever . ii 48

chronic ulcer of . . . vii 241

churning motion of . . ii 374

cicatrices of ulcers in . . xiii 517

complaints, air and exercise in vii 478

on alcohol in . . . xxiv 546

nux vomica in . . iv 244

concentrated saliva in . . xvi 221

congestion of mucous lining of xi 416

converting power of . . xi 332

cough, a source of . . . vii 128

digestion of, after death . . xv 93

of its coats after death . vi 180

digestive fluid in left half of . xvi 311

dilatation of, auscultation in . i 487

percussion in . . . i 487

diseases, constipation a cause of x 42

Dr. Alderson on . . xxiv 273

Dr. Seymour on . . xxiv 141

from spinal irritation . i 459

in army and navy . xvii 318, 321

disordered, reacts on teeth . ii 373

drinks in disordered . . vi 176

effects of excess on . . vi 176, 178

free living on . . . vi 177

empty, of animals, experiments on i 553

epithelium of . . . ix 567

exploration of . . . vi 139

external aperture in . . vi 173-74

*figure* of a child's . . . ii 539

of the adult . . . ii 540

free acid in . xviii 530, xxiv 273, 278

functions of, report on . . xvii 258

restored by galvanism . ii 137

fungoid disease of, case of . xxiv 142

fungous state of . . . xi 414

with cardiac disease . xi 414

glands of, action of . . xxiv 278

glandular membrane of . . vi 527

gleet, remarks on . . . xii 505

gradual development of . , ii 541

great enlargement of . . x 583

heat of, proper for digestion . ii 388

hemorrhage from ulcer of . vii 243

hydrochloric acid in . . xi 332

important remark on . . xi 417

inflammations of . . . xv 93

influence of food on form of . ii 541

inert, in the insane, treatment of ii 548

its influence on the sytem . ii 464

lactic or acetic acid in . . xi 332

mammillated state of . . xi 414

management of . . . vii 479

matter in tubuli of . . . ix 568

mischief of overtasking the . vii 123

morbid contraction of . . xi 413

states of . . . vii 115

thickening of . . . xi 414

Mr. L. Parker on ulcer of . vii 244

mucous membrane of . . ix 565

muscular coat of . . . xiv 546

Dr. Combe on ii 374

Stomach, natural colour of . xi 412, 415

nerves affecting . . . viii 269

nervous connexion of . . v 509

neurosis of . . . . xviii 208

of animals, diseases of . . xxiv 83

of human embryo, form of . ii 541

orifices of tubes of . . . ix 566

origin of azote in . . . xi 333

patent, Dr. Beaumont's case of ii 375

perforating ulcer of . . xiii 516

perforation of, by a worm . xiii 563

cases of iii 556, xiv 248, 577, xx 221

perforations of . . vii 243, ix 371

permanent acidity of . . xvi 221

-pump, injury from use of . xi 417

redundancy of . . . xi 413

regurgitation of . . . xx 238

revolution of contents of . vi 179

round, in diseased children . ii 541

saccharine principle in . . xi 332

secretes mucus only . . xvi 221

secretion of acid in . . vi 528-29

of gastric juice in . . vi 528

secretions of, alkaline . . xvi 221

sensibility of, in vomiting . ii 542

simple ulcer of . . . xvi 381

softening of . . . xi 417, xxiii 73

Dr Carswell on . i 138

two varieties of . xv 93

Dr. Dürr on . . i 257

spasm of, agreeable advice on . v 391

curious case of . . xxiii 267

spasmodic pain of . . . viii 357

spontaneous perforations of . vi 221

supplemental parietes of . xi 413

sympathetic influence of . vii 126

temperature of, remarks on . ii 377

ulcerations of, treatment of . xxii 469

ulcer of, causes of . . . vii 242

characters of . . . vii 242

cicatrization of . . vii 243

curable . . . . vii 241

description of . . vii 242

treament of . . . vii 242

ulcers of, cicatrization of . xxiii 73

undigested food in disordered . vi 176, 178

vegetable organisms from . xiv 247

vermicular motion of . . vi 179

view of cells of the . . ix 566

tubes of the . . . ix 567

vessels of the . . . ix 567

villous coat of, during life . vi 175

in disease . vi 176

whitish spots in . . . xi 415

Stomatoplastic, etymology of . . vii 408

operations . . . . vii 408

Stomatopoesis, operation of . . xxi 303

Stomatitis, and cancrum oris, distinct xvii 297

gangrene, contrasted xvii 297

diagnosis of . . . . xvii 556

different from thrush . . xxiv 428

in children, report on . . xvii 555

mercurial, the blood in . . xvii 145

Stomatitis, treatment of . . xvii 298
ulcerous, case of . . . xxiii 304
use of peppermint in . . xxii 54
Stone, and gravel, new remedy for . xiii 264
alkaline medicines in . . xii 390
benefit from Vichy waters in . xii 393, 397
chemical remedies for, war on xii 394
cures of, by Vichy waters . xii 396
dilating female urethra for . xii 401
effects of alkaline carbonates on xii 393
empirical remedies for . . xii 391
frequent in parts of Turkey . iv 396
high operation for . vi 169, xii 194-200
history of injections for . . xii 398
in bladder, case of . . . vi 69
frequent in India . . iii 330
Mr. Tyrrell on . . iii 149
remarks on . . . ii 98
Sir B. Brodie on . . xiv 165
children, frequency of . xiv 165
peculiar localities . . xiv 165
tonsils, case of . . . xi 528
lateral section for . . . xii 198
medicines by the mouth for . xii 390
new method of cutting for the ii 98
operations for at Naples . . xii 254
penchant for operations for . xii 389
-pock in the cow . . . ix 95
reflections on operation for . xii 389
removal of by solution . . xii 390
removed by Arnott's dilator . xii 407
spontaneous breaking down of xiv 166
treatises on . . . . xii 388
treatment of by injections . xii 398
Stool, a daily, errors respecting . vii 477
Stools, use of regularity as to . x 46
Stott, Mr., case of monomania in . x 137
Stourbridge, "Lye a waste" in . xxi 351
Stove, Dr. Arnott's thermometer . v 546
Stoves and open fire-places compared xix 17
Strabismus, accidents in operating for xi 491
alternating, myotomy in . xi 484
cause of failure in operation for xi 485
causes of . . . . xi 478
changes of muscles after operation for . . . . xi 489
convergens . . . . xi 476
alternating . . xi 477
double . . . xi 477
cured by operation . . . ix 558
without operation . xii 546, xiii 239
cure of, by operation . . x 585, 587
cutting recti of both eyes for . xi 485
dissection of divergent . . xi 479
dividing the abductor in . . xi 486, 487
divergent, operation for . . xi 488
double convergent, myotomy in xi 484
Dr. Brett on . . . . xxiv 371
Dr. Mackenzie on . . . xii 520
eye turned outwards after operation for . . . . xi 486
generally of only one eye . xi 476
M. Velpeau on . . . xv 240
Strabismus, morbid anatomy of . . xi 479
of left eye most frequent . . xi 476
operation for . . . . x 570-3
Dr. Ure on . . . xi 489
history of . . . xi 475
statistics of . . . xi 489
operations for, history of . xvi 67
remarks on . . . xviii 414
origin of operation for . . xii 564
principal forms of . . . xi 475
prominent eyeball after operation for xi 484
results of operation for . . xi 483
Sir C. Bell, on operations for . xi 492
state of muscles in . . . xi 479-82
statistics of . . . . xv 240
surnames from . . . xi 474
treatises on . . . xi 474, xxiv 364
Strait-waistcoats, use of, in St. Yon xix 606
Stramonium, endermic use of . v 349
on the fecula of . . . iii 229
poisoning by, case of . . xix 305
poultice of, in hernia . . iii 359-60
use of, in sarcocele . . . i 578
Strang, Dr., on instinct and reason xvii 525
Strangulated bowels, cases of . iii 495-500
hernia, Malgaigne on . xiii 245
new treatment of . vi 558
Strangulation, case of accidental . v 176
effects of . . . . xxii 161
in hernia, cause of . . . xxii 51
internal, intestinal . . xii 538, 551
intestinal, internal . . . iv 168
marks on neck from . . ii 419
mode of death in . . . xxii 162
of a child, case of . . . v 618
infants by the funis . . ii 415
on infanticide by . . . ii 415
parchment-like mark in . . ii 420
signs of death by . . . ii 419
suicidal, case of . . . vii 261
the tongue in death by . . ii 419
treatment of . . . . xxii 163
Strangury from blisters, obviating . xvi 205
Strasburg pâtés . . . . i 346
Streak, primitive, in the ovum . ix 18
Stream, a, delays putrefaction . ii 397
Street and house beggars, remarks on xxii 340
Streets and courts of towns, cleansing of xxi 339, 349
Streeten, Dr., his retrospective address xiii 520
on fever in Worcester . . vi 12
on influenza . . . . vii 95
Streeter, Mr., on abortion . . x 548
Stretching-bed, its use in scoliosis . v 143
Stricture, and spasm, belladonna in . ii 261
bellied bougies for . . . xvii 214
cauterization of . . . xvii 210
cure of, by caustic . . . xvii 213
not frequent . . . xvii 215
dilatation treatment of . xvii 209, 214
instrument of Ducamp for . xvii 212
Mr. Guthrie for . xvii 212
lancetted stilettes in . . i 534

Stricture, Mr. B. Cooper on . . xii 330

Mr. Guthrie on caustic in . xvii 212

Mr. Stafford's treatment of . xiv 581

near the prostate gland . vi 166

of colon, on non-malignant . xii 116

œsophagus, case of . . iii 73

Dr. Chelius on . ii 261

rectum, treatment of . . xxi 329

warning case on . x 45

trachea, case of . . . xv 153

urethra, armed bougies in . xiii 320

belladonna in . . ii 261

cause of . . . xvii 207

dilatation of . . xiii 217

pain of foot from . iv 133

seat of . . . xii 427

Sir B. Brodie on . xiv 160

spasmodic . . xiii 315

treatises on . . xiii 312

treatment of . . xiii 316

permanent . . . . xvii 207

of rectum, seat of . . iii 76

sonde exploratrice for . . xvii 208

spasmodic . . . . xvii 206

of the rectum . . . iii 76

treatment of . . . xvii 209, 215

urethral, report of cases of . xviii 341

Strictures, cicatrices in, treatment of xiii 326

gentle treatment of . . xiii 322

impervious, treatment of . xiii 321

nitrate of silver in . . . xiv 160

of urethra, on irritable . . xiii 315

model bougie for . xvii 208

new treatment of . iii 242

on examining . . xvii 208

situation of . . xvii 207

traumatic . . xiii 324

treatise on . . xxii 233

treatment of . . xix 521

sudden dilatation of . . v 572

traumatic, diagnosis of . . xiii 325

treatment of . . . xiii 325

urethral, classification of . xiii 315

on cutting in . . . xiv 161

gentleness in . . xiv 161

various modes of treating . xvii 215

Stridulous convulsion in infants . xxiii 197

Stromeyer, Guerin's depreciation of xiv 137

on operative orthopædia . . viii 384

on spinal curvature . . v 133

Strophulus, and lichen, species of . xv 391, 392

M. Rayer on . . . ii 154

Structural anatomy, history of . xiv 482

diseases, classification of . xvii 498

Dr. Williams on . . xvii 497

elements of . . . xvii 498

Structure, primary elements of . xvii 474

ultimate elements of . . xvii 474

Struma, Wiseman, on the royal touch for xxii 126, 151

Strychnia, action of . . . xvii 170

a cumulative medicine . . v 350

distinction of, from brucia . xvi 79

Strychnia, endermic use of . . v 349

experiments on its action . v 221

in capillary bronchitis . . xx 444

morphia, an antidote for . v 347

M. Rognetta's use of . . xvii 171

or bruise, separation of . . xvi 80

process for detecting . . xvi 79

Stilling's experiments with . xvi 423

tannin as an antidote for . xiv 229

tincture of, formula for . . xiii 84

use of, as an excitant . . xviii 528

Strychnine, M. Bally on the use of . vi 225

mode of action of . . xvi 419

restriction in the use of . . vi 225

use of, in neuralgia . . x 588

Stuart, his experiments on the breath i 243

Students, acquisition of knowledge by xvii 189

and practitioners, duties of . xvii 188

English, at Paris, book for . i 501

future success of . . . xvii 190

London medical, remarks on . ii 473

manuals for, remarks on . i 512

medical, on examination of . ii 478

health of, in Paris . . v 455

in Copenhagen . . ii 286, 288

Norway . . . iv 542

the United States . ii 593

number of, in Prussia . i 296

registration of . . xix 549

military, medical, in Prussia . i 297

on continent, book for . . i 500

medical map for . . i 500

real difficulties to . . xvii 191

self-instruction by . . xvii 190

surgical, Hindu, teaching of . xxiii 539

their present conduct . . xvii 189

Studies, medical, moral effect of . xxi 441

Study, benefit of change of . . iii 475

energetic devotion to . . xxiii 243

medical, order of, at Bologna . i 491

Sir B. Brodie on . . xxii 160

Stupor, in typhoid fever, its origin . ii 47

Sturgeons, spines of, Prof. Owen on xxiii 482

Stuttering, cerebral and spinal . xx 556

cure of . . . . . . xii 209

occasioned by worms . . iii 231

Stuttgard Selections in children's diseases . . . . ii 353

Style, in medical literature . . xxi 445

of medical works, remarks on . xii 185

writers, remarks on v 393

remarks on, and specimen of . iv, 251, 252

Styptic, matico, a new . . . xvii 100

Styptics, in hemorrhage, hazard of . x 66

remarks on the use of . . xii 441

Styria, sanitary statistics of . . xix 374

Subclavian arteries, deviations of . xix 490

artery, aneurism of . . xxiv 420

ligature of . . xix 497, xxiv 322

left, deviations of . . . xix 495

right, branches of . . . xix 491

Subclavicular region, prominence of vii 366

Subcutaneous division of cicatrices . xxi 290

Subcutaneous fooleries, Dr. Pauli on xxii 50

incision . . . . . . ix 554

operations, M. Guerin on . xiv 136

multiplied . . . xiv 139

section, history of . . . xvi 66

sections, by M. Guerin . . xi 250

tenotomy, rapid strides of . xiii 23

wounds, experiments on . . x 265

Sub-inflammation, Dr. Lanza on . xxiii 55

Subjectivity of disease . . . xiii 260

Sublimate ointment, Dr. Romberg on xxiv 45

Submammary region, on dullness of iv 294

Submaxillary duct, case of calculus in ix 248

gland, extirpation of . . xiii 238

Submersion, action of the heart in . xxi 269

Subserous tissue, inflammation in . iv 46

intestinal lacerability of iv 51, 52

Subsistence, on the problem of . xvi 229

Substitutions, law of, in chemistry xi 147

Subsumption of disease . . . xx 317

Success of medical men, secret of . xvii 191

Succession, rights of . . . ix 44

Successor, Editor's advice to his . xxiv 598

Succussion, sound on, diagnosis from vi 267

Sucking, by anencephalous puppies v 507

fish, median ganglion of . . xxii 498

in the infant, nature of . . v 506

Suckling, advantages of early . x 124

benefits of, mutual . . x 125

by males . . . . . x 111

virgins and old women . x 111

children, cases of men . . vi 79

food of the mother during . x 126

on regularity in . . . xix 71

obstacles to mothers . . x 195

Suckow, Dr., his useless work . vi 147

on semeiotics . . . vi 128, 147

Suction-pump, in strangulated hernia i 586

Sudamina, description of . . ii 37

in typhoid fever . . . ii 37

Sudden death, causes of . . x 424

deaths, in England, remarks on ix 356

Sudorific glands, in mammalia . vi 514

medicines, Dr. Holland on . viii 490

process in hydropathy xxii 438-9, 450

Sudoriparous glands of the skin . xv 380

Suetin, M., his treatment of fractures vi 314-5

Suffering and self-consciousness . xxi 105

Suffocation, by plugging the throat ii 414

from polypus, case of . . iii 72

Sugar, and carbonic acid, formulæ of xxiii 502

an alimentary principle . . xvii 8

as an improver of the blood . xiv 419

butyric acid obtained from . xvii 15

case of dyscrasy treated with . v 243

-conversion in the stomach . xi 338

digestion of . . . . xxi 562

Dr. Prout's opinion of . . iv 26

formation of, in diabetes . xiv 510

found in the blood in diabetes ii 281

in cutaneous eruptions . . v 243

diabetic blood . . . xvii 432

Mr. Jones on . xviii 369

Sugar, in food, disposal of . . xxiii 393

in healthy stomach . . vii 165

system, observations on . xxiii 501

urine, yeast the best test of vii 166

mal-assimilation of, causes of . xi 339

normally in the stomach . xi 338

nutritive power of . . xiii 490

of the food, alcohol from the . xxiii 160

relative sweetness of . . xvi 199

starch, and gum, similar . xiv 304

&c., use of in food . xiv 304

transformations of, in food . xix 563

Suicidal disposition, treatment of . xii 152

Suicide, acute and chronic . . xx 26

an act of insanity . . . v *379

anatomy of . . . xii 149

at the present day, M. Pinel on xx 26

case and dissection of a . . v 377-8

case of, Mr. Doherty's . . vi 473

compassion for the . . . v 379

continental, statistics of . . iv 234

cowardice of . . . . xii 150

curious case of . . . xiv 583

attempted . . vi 474

by a barber . vi 171

delirium sine febre . . v 369

French and English law in . x 169

from blind impulse . . xii 150

feeble-mindedness . . xii 152

perverted instinct . . xii 152

strangulation . . vii 261

hereditary disposition to . v 373

in Aberdeen, statistics of . iii 270

an asylum, remarks on . xix 607

botanists, Osiander on . v 374

England, chief cause of . v 371

remarks on . . iv 334

statistics of . . xv 76

France . . . . . x 162

statistics of . . xiii 552

gastroperiodynia . . iii 216

Geneva, M. Prevost on . ii 277

statistics of . . . ii 277

London, statistics of . . vi 241

relation to education . . xv 77

Russia and Germany . . v 371

Spain, prevention of . . v 371

the capitals of Europe . vi 241

means of preventing . . xv 78

months favorable to . . xii 151

morbid appearances in . . v 373-7

M. Cazauvieilh on . . . x 129

not always from insanity . xvi 86

number of deaths by, in Ireland xviii 44

in various cities . xxi 214

on publishing cases of . . xv 78

periodical occurrence of . . v 373

phrenological idea of . . v 380

propensity to, in lunatics . xi 120

rare in Italy . . . . v 371

remarkable cases of . . xiv 257

singular modes of . . . v 369

Society of the Friends of . v 371

Suicide, statistics of . . . v 371
table of the means of . . v 370
tables in relation to . . xviii 176
the result of insanity . . xii 153
time of committing . . v 371
treatises on . . . . . v 368
trifling causes of . . . v 370
when not from insanity . . xv 425
Suicides, in different professions . i 198
in England, in 1840 . . xxi 3
Sulphate of copper, with tartar emetic xv 73
potash, poisoning by . xviii 556
Sulphatoxygen, a compound radical xi 140
Sulpho-cyanic acid, poisoning by viii 258, 260
-cyanogen, in the saliva . . xiii 260
Sulphur, as an element of food . xvii 3
-baths, in cutaneous diseases . i 497
carburet of, in rheumatism . ii 252
in rheumatic affections . . x 586
spasmodic affections . . x 586
milk and flowers of . . viii 354
Sulphuret of carbon, a dog poisoned by i 245
lime, in skin diseases . iii 242
Sulphuretted hydrogen, in air . xix 14
in lead colic . ii 575-6
Sulphuric acid, absorption of . vi 244, 246
arsenic in . xiii 192, xviii 542
cases of poisoning by . iii 539, ix 391, xiv 584
wounds by . iii 533
effect of, on blood . viii 345
swallowing . xi 417
free, on detecting . xvi 78
poisoning by . . xx 327
pure, springs of . . viii 359
of shops, arsenic in . xiv 123
Sulphurous acid in air . . . xix 14
bath, composition of . . i 497
perspiration, case of . . xii 192
waters . . . . . . xiv 327
*Sume superbiam*, complacency with xvi 490
Summer most fatal to children . xxiv 112
Sun, the, magnetised by Mesmer . vii 314
Sunbeams, action of, on plants . xx 155
Sun-light, salutary influence of . i 336
Sun-stroke, on cerebral affection from iii 305, 310
Sunday observances in schools . v 403
Superfœtation, Prof. Devergie on . ii 405
remarks on . . . . . ix 43
Superintendent, medical, of an asylum xxiv 164
Superstitions, medical, Mr. Pettigrew on xviii 234
Suppers, disapprobation of . . xvi 217
Suppressed perspiration, causes of disease of kidneys . . . ii 226
Suppuration, causes of . . . xviii 111
diffused, explanation of . . xviii 112
Dr. Carswell on . . . i 148
Mr. Gulliver on . . . vi 553
Mr. T. W. Jones on . . xviii 275
not a healthy process . . xviii 75
phlegmonoid . . . ii 24
unbounded, destructive . . ii 24
Suppurative erysipelas, Mr. Travers on ii 24
Supra-condyloid processes . . xix 571
-renal capsules . . . vii 241
Surface-treatment, in hydropathy . xxii 442
Surfeit of plums, effects of . . xi 232
Surgeon, military, his duty at punishment . . . . . xxiii 164
scientific, of the present day . xiv 407
the natural, of Hunter . . xxiii 588
Surgeons' diplomas in Ireland, number of, in ten years . . i 610
Vademecum, Mr. Druitt's . viii 536
Surgeons, and physicians, education of xiv 407-8
College of, in London . . xiv 407
Italian, who give no medicine . i 498
London College of . . xii 576
military, authority of . . xviii 123-4
duties of . . . xviii 123
native, in India . . . xix 74, 78
naval, advice to . . . xviii 221
of Prussia, their examination . i 301
Surgery, almost 'tabooed' by the Koran iv 395
and medicine, anciently united xiv 406
one science . . . vi 98
relations of . . xiv 402
application of mesmerism in . xxii 475
as a science and art . . xx 461
as it was and is . . . xii 389
autoplastic, cases in . . ix 559
Chelius's Manual of xi 305-6, xx 110, 119
clinical, Lisfranc's xiv 1, xv 30, xviii 1, 35
Mr. Ellis on . . . xxiii 250
of Strasburg . . . x 49
Cyclopædia of . . ix 539, xiii 168
Dr. Colles' lectures on . . xxi 271
Dr. Pauli's observations on . xxii 49
Dr. Von Walther's system of . ii 202
etherization in . . . . xxiii 556
is it a science? . . . vi 103, 106
of the ancient Hindoos . . xxiii 538
masters, in history of . . xiv 405
military, Dr. Ballingall on . ix 243
M. Lisfranc's clinical . . xviii 1, 35
M. Moulinié's success in . xvi 266
Mr. Cocks's Operative . . v 544
Mr. Fergusson's Manual of . xxi 483
Practical . . . xv 188
Mr. Liston's Elements of xi 305-6, 321
lectures on . . . xxii 379
Practical . . . vii 235
Mr. Lizars's System of . . x 537
Mr. Morgan's Principles of iv 185, x 532
Mr. Syme's Principles of iii 481 xiv 211
M. Vidal on . . xi 305-6, 321
on make-shift . . . iv 232
Operative, Dieffenbach's xxi 285, 333
Dr. Pancoast's . . xx 39
M. Malgaigne's . . xxiii 155
Parisian, Dr. Markham on . xi 218
plastic, definition of . . vii 387
Dieffenbach on . . xxi 292
Dr. Zeis's Manual of . viii 384
nomenclature of . . vii 388
treatises on . . xix 396

Surgery, plastic, works on . . vii 386

Practical, Mr. Liston's . . xxii 366

treatise on v 457

Mr. Lizars's . . . vii 231

principles of . . . xix 524

Professor Müller's . . xxi 488

relation of, to medicine . . xvi 507

remarks on exclusive . . ix 228

scientific, advancement of . iv 185

some of the triumphs of . xxii 559

Sir B. Brodie's lectures on . xxii 160

Sir C. Bell's Institutes of . vi 154

state of, in Turkey . . iv 395

study and practice of . . xx 111

triumphs of . . . . . vi 257

Surgical affections, Walther's classification of . . . . ii 203

anatomy, treatises on . . x 347

cases, by Mr. B. Cooper . vi 66

copper-plates, by Dr. Froriep . iv 168

Dictionary, Mr. S. Cooper's . xi 305-6

diplomas, at Edinburgh, 1835-6 iii 292

at London, 1835-6 . . iii 292

diseases, in India . . . xii 422

use of cold water in . iii 114-28

eccentricities, M. Mayor's . xx 461

improvements, remarks on . viii 385

instruments, gilding of . . xiv 233

literature, retrospect of . . viii 546

miscellanies, Dr. Kerst's . . xv 399

observations, by Prof. Chelius ii 259

operations, causes of death in . xviii 333

history of . . . xvi 46

Lisfranc on . . . xv 30

painless by ether . . xxiii 309

remarks on . . . . xx 111

pharmaceutical studium in Berlin i 298

report of Glasgow infirmary . iii 558

Surveyors for drainage works required xxi 337

Susceptibility to disease, Dr. Marx on xvi 475

Suspended animation, galvanism in x 584

Suspension, during life, new signs of viii 572

sure signs of ii 421

signs of . . . . . xii 270

Susruta, on ancient Indian medicine xxiii 521

diseases treated of, in the . xxiii 542

subjects in the work of . . xxiii 531

Suttees, effect of civilization on . xviii 244

Suture, of the palate, Dr. Smith on i 265

subcutaneous . . . . xxi 286

Sutures, and fontanelles, Dr. Dewees on ii 76

their importance in labour ii 76

caoutchouc threads for . . xii 282

for wounds of intestines . xxiii 9, 16

observations on . . . vii 399

Swaine, Dr., his Translation of Hasse xxiii 251

Swainson, Mr., on instincts of animals xi 90, 103

Swammerdam, his discoveries . xiv 483

Swamp, one in St. Lucia described . iv 161

Swan, Mr., his fondness of theory . x 511

his illustrations of anatomy . iii 489

illustration of the nerves . ii 192

observations on deafness . iii 83, 100

Swan, Mr., on the brain and nerves xviii 532

nervous system . viii 226, x 508

sympathetic nerve . xxiii 582

Swansea, effect of gaseous products in xxi 353

Sweat, composition of . . . xvii 443

Sweat-glands, number of . . xix 567

Sweat, hectic night-, treatment of . xix 23

said to be purulent . i 509

variations of in diseases . . xvii 443

Sweating, cure of diseases by . xxii 450

dry mode of producing . . xxii 439

miliary epidemic fever . . vii 244

Osiander on puerperal . . iv 399

process in hydropathy xxii 438, 439, 450

sickness, epidemic of . . xiii 534

in India, account of . xiii 355

miliary epidemic . . xviii 162

state of blood in . . xiii 356

symptoms of . . . xiii 355

treatment of . . . xiii 356

Sweatman, Dr., obituary of . ix 296

prompt cure by . . . v 389

Sweats, local, observations on . xx 312

Sweden, mortality in, referred to . iv 262

Swedes, form of their skulls . . xviii 373

Sweetmeats, use of, after meals . xvi 223

Sweetsir, Dr., on mental hygiene xvi 511

Swera, the first physician in Russia i 598

Swine-pox, Jenner's experiments on vi 479

Sycosis, a severe form of . . ii 152

M. Rayer's description of . ii 151

Sycosis menti, treatment of . . xx 268

Sydenham, on typhus fever . xii 295

remark of Dr. West on . . xv 564

works of, by Dr. Greenhill . xviii 526

Sydenham Society, progress of the xvii 522

prospectus of the . . xv 573

publications of xviii 526, xx 206, xxiii 251

Sydney, New South Wales, diseases at v 261

Syllabus of lectures on nervous system i 203, 204

Sylvius, father of chemical physiology i 383

Sylvius, fissure of, in the brain . xviii 435

Symblepharon, treatment of . . xxi 309

Syme, Mr., his Principles of Surgery iii 481, xiv 211

omissions in . . xiv 212

on diseases of the rectum . v 480

the periosteum . . . viii 285

unjustly accused of plagiarism vii 438

Symeles, monsters so called . . viii 30

Symmetrical diseases, papers on . xv 149

Symmetry of disease, remarks on . xxiii 136

law of, in vascular lesions . vi 49

Symonds, Dr., arrangement of diseases by . . . . . x 234

Dr. A. Combe in reply to . xxiii 257

his address at Liverpool . . x 461

in reply to Dr. Combe . . xxiii 596

on gastritis . . . . xii 115

knowledge . . . xxiii 252

medical study . . . iii 201

on trust in Nature . . xxii 557

Sympathetic actions, disquisition on iii 33

Dr. Whytt on . iii 33

Sympathetic actions, production of . xxiv 193

various authors on . iii 33

acts, on expression of . . xviii 448

fibrils, origin of . . . xvii 385

ganglia, office of . . . xi 298, 304

reflex function of . . xxiv 204

motions . . . . . xxiv 311

nature of . . . v 522, 537

nerve, Dr. Procter on . . xviii 528

effects of, on the blood . xviii 528

experiment on . . xviii 528

Mr. Swan on . . . xxiii 582

functions of . . . v 520

on tying . . . iii 155

organic fibres of . . vii 501

origin of . . . xiii 344

origin of, *figures of* . i 562-3

traced . . i 560

relations of . . . xvii 401

source of its power . . ii 133

structure of . . . vii 541

nerves, and brain, connexion of xxii 406

colour and softness of . xvii 382

connexions of . . . xvii 383

fibrils of . . xvii 381-86

hyperesthesiæ of . . xvii 415

report on . . . xvii 268

nervous fibres, independent . xiv 296

system . . . . ix 103

remarks on . . ii 133

phenomena, practical use of . iii 39

remarks on . . . viii 355

sensations, examples of . . xxiv 311

system, anatomy of . . xvii 380

centres of . . . xxii 274

independence of . . xix 576

inference on . . . viii 511

none in invertebrata . viii 511

Sympathies, acquired abnormal . ix 407

abnormal . . . . xxiv 314

anormal nervous . . . ix 406

anormally increased . . ix 406

classification of . . . ii 181

morbid, division of . . ix 458

transmission of . . ix 457

nervous, Dr. Henle on . . ix 404

normal . . . . . xxiv 307

of cellular tissue . . . xxiv 312

cerebro-spinal nerves . . xxiv 308-9

nerves of vessels. . . xxiv 312

nerves, Whytt on . . xix 99

the sensorial organ . . xxiv 313

unknown origin . . . xxiv 314

visceral nerves . . . xxiv 309-12

old theory of particular . . vii 70

through the blood . . . xxiv 307

nerves . . . xxiv 307

Sympathisers, on awkward . . xviii 473

Sympathy, absence of, in medical works xxi 445

and antagonism . . . xxiv 306

metastasis the same . . ii 8

definition of . . . . xxiv 307

diseases from . . . xii 67

Sympathy, Dr. Alison's paper on . v 533

Dr. Philip's views of . . ii 138

great need of, to women . xviii 473

in relation to sensation . . iii 34-9

spinal cord . iii 34-9

John Hunter on . . . v 50

Mr. Macilwain's theory of . ii 180

nervous, first truly explained . i 384

organic, Dr. Fletcher on . v 97

phenomena of . . . iii 33-9

physiology of . . . ii 139

the cause of disease . . ii 181

Van Helmont on . . . xvi 411

Symphisis pubis, disjunction of . xx 532

Symphoresis, or congestion explained iii 209

Symphyses, congenital, of M. Breschet viii 12, 14

Symphysotomy, remarks on . . ix 39

report on . . . . xx 534

Symptomatic and idiopathic disease xxiv 305

Symptomatology, in diagnosis . xx 309

remarks on . . . . vi 303

Symptom, signification of the term iv 289

Symptoms, and signs, distinction of xvii 123

remarks on . xvii 499

operations, Galen on xiii 208

arrangement of . . . xx 317

combination of, in disease . xxiii 42, 50

Lanza on . xxiii 50

intensity of . . . . xii 14

many from opposite causes . iii 223

mode of investigating . . iv 289

studying, by M. Louis i 400

morbid and curative . . xiii 207

of disease, Schönlein on . xxi 21

diseases, remarks on . . xii 13

the medicine of, remarks on . xiv 140

threefold view of . . . iv 289

vital, Dr. Williams on . . xvii 499

Synchondroses, pelvic, destruction of xiii 118, 171

Syncope, alarming, from air in veins xx 87

and apoplexy, not allied . . xxii 416

case of slow pulse in . . xiv 55

cause of insensibility in . . xvii 352

curious arrestment of . . v 147

from nervous injuries . . xviii 214

from tap on the spine . . xii 106

in old age . . . . xvii 110

rationale of . . . . iii 325

various causes of . . . xviii 216

Synechia, anterior and posterior . xx 285

Synergy and antagonism, in sympathy xxiv 306

Synopsis of diseases, new . . xiii 202

Synocha, in India, treatment of . iii 349

purest example of, in India . iii 349

Synovial and serous sacs, analogy of x 331

cavities, M. Velpeau on . . xviii 81

effusions, emetic tartar in . vi 224

membrane, a shut sac . . xiii 169

inflammation of . x 333, xx 113

thickening of . . . x 334

membranes, diseases of . . x 331

suppuration of . . x 336

sacs, adhesions in . . . x 335

Synovial sacs, dropsies in . . x 331
tissue, peculiar disease of . x 335
tumours, new treatment of . ix 554
Synovitis, M. Richet on . . xxi 127
Syphilides, the, in baths . . xxiii 371
common symptoms of . . xxiii 352
fumigation in . . . xxiii 371
history of . . . . . xxiii 350
primary and secondary . . xxiii 363
proto-iodide of mercury in xxiii 366, 370
special symptoms of . . xxiii 352
time of appearance of . . xxiii 363
treatment of . . . . xxiii 364
various remedies for . . xxiii 369
Syphilis, and gonorrhœa, identity of x 384, xxiii 347
animate contagion of . . xi 219
arising spontaneously . . i 371
a specific disease . . . v 16
black oxide of mercury in . v 18
Carmichael on mercury in . x 381
case of, mistaken for plague . xxiv 258
cause of, a morbid poison . v 16
Cazenave on the treatment of . xxiii 349
chlorurets of mercury in . v 18, 19
coexistent with plague . . xix 46
complications in, causes of . ii 19
congenital . . . . . v 15
constitutional . . . . x 412
effects of . . . v 11
contagious property of . . v 16
continuance of mercury in . v 24
cura famis of, in Sweden . v 5
cura magna of Dr. Bene in . i 18
decoction of Zittman in . . i 18
diversity of forms of . . v 8
Dr. Bene on . . . . . i 16
mercury in . . i 17
dry course of mercury in . v 23
effect of civilization on . . xviii 248
efficacy of gold in . . . v 20
endemic in Lithuania . . vi 223
experiments on . . . . v 4, 6
fatal, without external sign . ii 18
French Council of Health on . v 5
from vaccination . . . xx 563
guaiacum in . . . . . v 20
Hamburg reports on . . v 5
hydrargyri iodidum in . . x 414
hydriodate of potash in . v 20, vii 245
in ancient times . . . xxiii 346
infection of nurses by . . v 15
influence of season on . . xxiv 104
in French army, prevention of xv 282
Hungary . . . . . i 17
India . . . . . xix 73
inoculation of . v 10, 16, x 382, 387
by infants . . x 388
in Parisian prostitutes . . iv 74
pregnant women . . xii 541
interesting case of . . xiv 578
in the Enfants Trouvés . . vii 93
iodine in . . . . . v 20
its early and late history . v 3
Syphilis, masked, opinions on . v 13
mercurial ptyalism in . . v 22, 23
treatment of iv 74, v 18, 21, ix 240-1
mercury in, Dr. Colles on . v 22
remarks on . . v 21
Sir B. Brodie on . xxii 169
mode of curing by iodine xix 516, 522
modern treatment of . . xxi 495
Moj'sisovic's pathology of . xix 520
M. Cullerier's practice in . x 418
M. Ratier on . . . . . xxii 226
Mr. Parker, on treatment of . ix 239
natural history of . . . v 7, 16
neonatorum, remarks on . . xxi 468
non-mercurial treatment of . v 4, 7, ix 219, 240-41, xv 468
on a double virus in . . xxiii 348
specific virus in . . v 8
phagedenic, primary forms of . x 405
platina, a remedy for . . xi 523
primary, propositions on . xxiii 346
treatment of . . x 388, xix 521
progress of . . . . . v 16
relapses after simple treatment of v 20
remarkable case of . . . xxiii 366
salivation in, by Dr. Bene . i 18
sarsaparilla in . . . . v 20
secondary, Dr. Colles on . v 12
Hunter on . . . . v 11, 13
infectious . . . . v 16
hydriod. potass. in . . iii 561
inoculation from . . . x 387
iodide of potassium in . x 415
prevention of . . . x 413
remarks on . . . . v 11
mercurial treatment of . x 414
symptoms of . . . . x 413
treatment of . v 19, 24, x 414, xix 521, xx 486
simple treatment of . . v 17
Sir C. Bell on mercury in . vi 171
successive . . . . . x 413
three different stages of . . x 412
treated by rigid abstinence . i 500
without mercury . i 17, 500
treatises on . . . . . xii 362
treatment of . . . v 17, 25, vi 172
by iodine . . . xx 482
of, in France . . ix 240
tubercular forms of . . . x 416
various treatises on . . . v 1
with scrofula, mercury in . v 25
Syphilitic affections of animals . xxiv 89
the bones . x 416
bullæ and pustulæ . . xxiii 355
cachexia, signs of . . . ii 20
diseases, treatises on . . x 381
eruptions . . . . . v 13
causes of . . . xxiii 362
Cazenave on . . . xxiii 345
classification of . . xxiii 353
colour of . . . xxiii 351
diagnosis of . . . xxiii 364

Syphilitic eruptions, treatment in . v 25

infection in labour . . . viii 53

life, statistics of . . . xxiii 364

muscular retraction . . xiii 543

nocturnal pains in bones . . x 416

origin of rheumatism in India . xxiii 127

papulæ . . . . . . xxiii 360

phagedenic ulceration . . xii 339

poison, modification of . . xii 337

poisons, diversity of . . xii 336

primary sores . . . v 14, 16

treatment of . v 23

sores, inoculation from . . xii 373

primary, mercury in . x 398

treatment of . x 398, 401

varieties of . . xii 335

squamæ . . . . . xxiii 361

temperament of M. Ricord . x 413

tertiary symptoms . . . x 413

tubercular eruption, varieties of xxiii 358

tubercle, characters of . . xxiii 357

tubercles, treatment of . . x 415

ulcerated throat . . . v 13

ulceration of the larynx . . xviii 295

ulcers, use of leeches in . . ix 240

vesiculæ . . . . . xxiii 355

virus, Mr. Judd's theory on . v 9

Syphiloid affections, remarks on . v 8

cachexia, bath for . . . iv 254

diseases of animals. . . xxiii 347

Syphon catheter in lithotomy . xii 194

Syren group, two auricles in heart of xv 208

Syrens, or Symeles . . . viii 30

Syrian animals, long white hair of . iv 130

Medical Aid Association . xiii 579

Syringe, improved female . . v 307

Syringes for, and syringing, the ear iii 89

Syro-Arabian languages . . xxiv 455

nations . . . . . xxiv 454

Syrup of poppies, frequent death from iv 118

important remarks on . iv 118

Syrupus papaveris, adulteration of . iv 118

Systems, in medicine, value of . xix 371

medical, of Germany . iii 451

nosological, origin of . xxiii 97

Systole of auricles, a notable impulse in i 448

heart, prolongation of . i 488

Tabaci enema, properties and use of iv 114
Tabes mesenterica, diagnosis of . xvii 311
in children . . xvii 311, 560
Irish notions of . . xviii 40
pathology of . . . ix 479
Table of income and expenditure of
the Review . . . xxiv 596
Tables of cardiac lesions in apoplexy xxii 421
zoochemical substances . xxiv 286
Tachytomie, art of cutting rapidly . xx 463
Tact, medical, remark on . . xiv 437
Tactile vibration in pericarditis . xxiv 553
Tadpoles, on globules in lymphatics of iv 500
the transparency in veins of vi 219, 220
Tænia, pomegranate-root bark in . iv 112
lata and solium . . . xviii 320
solium, account of . . xx 418
found in ditch-water . xx 418
Tahitians, physical characters of . xxiv 468
and Marians, complexion of . xxiv 473
Tail, deficiency of, in carnivora . viii 6
of lizards, duplication of . viii 24
Tailors, observations on . . xv 335
Tailors' workshops in London, gin in xxiv 547
Taliacotius, Butler and Voltaire on vii 389
his plastic operations . vii 388, 390
rhinoplastic operation . vii 390
work on plastic surgery vii 388, 390
or Tagliacozzi . . . vii 388
Talipes calcaneus, double, cure of . xiii 12
equinus, causes of . . viii 395
description of . . viii 394
operation for . . viii 396
valgus, cause of . . . viii 398
description of . . viii 395
operation for . . . viii 398
varus, description of . . viii 395
cured by operation . . vi 272
Talipes, cause of varieties of . . xiii 24
complications with . . xxi 183
opinions on the cause of . xxi 400
treatment of . . . xxi 182
Talma, his death from stricture of
rectum . . . . ii 331
unsuspected aneurism of his heart ii 331
Tampon, in uterine hemorrhage, use of x 98
Tamulian race of North Ceylon . xxiv 462
Tan-baths, mode of forming . . xvii 378
Tannic acid, preparation of . . xiv 440
Tannin, as an antidote for strychnia xiv 229
solution of, used in chancre . x 403
tincture of, in leucorrhœa . xxiii 293
Tanquerel, M., on diseases from lead x 426
Tapetum, on the office of the . xxii 285
Tapeworm, the, age of . . . xviii 321
and dysentery, bread-pills in . xxiii 267
cure of . . . . . iv 112
discrepant opinions on . . xviii 325
diagnosis of . . . . xviii 322
Dr. Wawruch on . . . xviii 319
his treatment of xviii 328-32
etiology of . . . . xviii 323
exciting causes of . . . xv.ii 324
experiment on the growth of . xviii 321
hereditary nature of . . xviii 323
influenced by soil . . xviii 321
in relation to ague . . xviii 324
is hermaphrodite . . . xviii 320
local peculiarities of . . xviii 321
mode of growth of . . xviii 321
persons most subject to . . xviii 324
principal seat of . . . xviii 321
prognosis of . . . . xviii 326
terminations of . . . xviii 325
Tapping, in ascites, Dr. Seymour on iii 438
in ovarian dropsy . . . iii 139
the head, in hydrocephalus . vi 265

TARAMELLI, Dr., on hernia of appendix cæci . . . i 583

Tarsi, Dr. Zeis on the . . . v 218

on their use in man . . v 219

Tarsus, dislocation of . . . vi 538

luxation of, tenotomy in . xiii 21

new mode of amputating . x 271

Tarsorhaphie, operation of . . xxi 308

Tartar-emetic, action of, in pneumonia xx 81

and opium, in fever . xvi 238

use of . . . v 275

friction, M. Polli on . viii 251

in affections of the head . ii 146

pneumonia . . xiii 409

puerperal convulsions . ii 87

synovial effusions . vi 224

mechanical action of . viii 251

ointment, in uterine disease ii 517

Jenner's . . iv 119

strength of . . iv 119

peculiar use of . . vii 125

poisoning by . . xviii 552

Tartaric acid, large doses of poisonous xx 328

Tartarised antimony, preparation of xiii 60

tests of . . . xiii 61

Tartars, observations on . . xxiv 383

Tartrate of antimony, clyster of . xv 73

in head affections . ii 146

Odier's use of . . iii 458

Tar-water, in psoriasis, use of . xxiv 48

in pulmonary affections . iii 509

preparation of . . xx 449

Taste, and smell, nerves of . . xvii 143

discussion on the nerves of . v 110

Dr. Alcock on the nerves of . iii 259

glosso-pharyngeal, the nerve of ii 600

impaired, in cerebral tumours iv 378

loss of, in facial paralysis . xxii 182

nerves of the sense of . . xi 288

Taurine, chemical composition of . xiv 517

TAVERNIER, Dr., on spinal deformity xvi 347

TAVIGNOT, on Pott's disease of spine xxi 124

Tawell, absurd defence of . xx 333-4, 337

Taxis in hernia, new mode of . vi 558

proper line for . . xv 40, 41

remarks on . . vi 559

warning case of . . xxii 199

TAYLOR, Dr. John, on cancer of the lung xv 430

on causes of pericarditis . . xxiii 415

pericarditis . . . xxiv 548

TAYLOR, Dr., W. C., his tour among factories . . . . xv 285, 309

TAYLOR, Mr. A. S., a case of poisoning by xiv 122

cases of poisoning by . xviii 332, 342

his Manual of Jurisprudence . xxi 484

medico-legal cases . . vii 454

report on toxicology . . xviii 533

thermometrical table . xix 562

on arsenic . . . . . xii 342

arsenical poisoning . . iv 358

infanticide . . . xvi 309

medical jurisprudence ii 390, 422, xvii 228, 232, xxiii 423

perforations of stomach . ix 371

TAYLOR, Mr., on poisoning by arsenic xxiii 425

opium . . xxiii 416

on temperature of the earth . xxii 229

test for arsenic . . . xvi 275

the lungs of infants . . vi 62

urea in the blood in fever . xviii 179

water and lead . . . vii 448

Tea, constituents of . . . xvii 21

and coffee, food for the liver . xiv 519

salubrious effects of . xiv 517

best time for drinking . . ix 242

common, of the lower classes . ix 242

green, evil effects of, on some . ix 243

indefinite use of the term . ix 242

injurious, in weak nerves . xiv 359

medicinal and moral effects of ix 241

professional opinions on . . ix 241

Teachers, medical, responsibilities of xvii 122

of anatomy, and surgery . ii 610

regulations on . . iii 292

Teaching, Count de Tracey on the art of . . . . . i 361

TEALE, Mr., on abdominal hernia . xxii 193

Tears, of infants, Billard on . . ii 356

remarks on . . xi 201

Rognetta's notion on the . x 13

Teaspoon swallowed, case of . . v 252

Teeth, the, affected by temperature ii 373

animals classed by structure of x 209

arrest of development of . xxi 68

a test of age . . . . v 205

blastema of the capsule of . xx 390

bone of, structure of . . xiv 270

cells of, pulp of . . . xii 491

cementum or crusta petrosa of xx 380

comparative anatomy of . x 208

contents of tubes of . . viii 161

crusta petrosa of, structure of . xiv 270

cutting of, in old age . . vi 80

decayed, cause of . . . xvi 223

development of viii 165, xii 491, xx 383

cementum of . . viii 168

enamel of . . viii 167

discoveries on structure of . viii 159

enamel of . . viii 163, xx 381

structure of . . . xiv 270

exostosis of . . . . ix 444

extraction of, for tic douloureux xxii 170

fibres or tubes of . . . viii 160

formation of crusta petrosa of . viii 168

formed by calcareous deposit . x 209

fossil, on the structure of . xxi 70

grow by intussusception . x 209

human, peculiar substance on . xi 533

in relation to digestion . . ii 373

intertubular substance of . viii 162

investments and pulp of . ix 443

in Warwickshire sandstone . xx 404

ivory, or bone of . . . viii 160

of, formation of . . xx 378

structure of . . . xiv 270

minute structure of . . ix 513

Mr. Goodsir on . . . vi 583

Mr. Nasmyth on the ivory of . xii 492

Teeth, not extravascular . . x 209

of fishes, great variety in . xx 391

mammals, Prof. Owen on . xxi 60

succession of . . xxi 67

reptiles . . . . . xx 398

development of . . xx 402

origin and development of . vii 571

ossification of pulp of . . ix 444

parietes of tubes of . . viii 161

*plate of*, explanation of . . viii 168

Prof. Owen on the structure of vi 578

pulp of . . . . xii 491

or germ of . . . viii 166

Retzius, M., on . . . viii 158

structure of . . vii 276, x 209

*plate of* . . . . viii 158

report on . . . xvii 257

termination of tubes of . . viii 163

treatises on the structure of . viii 158

treatment of . . . . ii 373

use of the tubes of . . . viii 162

Teetotal experiment, by Dr. Beddoes xxiv 539

suicides . . . . xxiv 545

Teetotallers, brickmaking by . . xxiv 535

Teetotalism, high medical authority for xxiv 516, 522

ignorant enthusiasts in . . xxiv 523

of a gigantic workman . . xxiv 539

on sudden or gradual . . xxiv 547

on medical attention to . . xxiv 515

Prof. Schultz on . . . xvi 221

structures on . . . viii 528

Teevan, Mr., on black secretion from skin xxiii 416

Teheran, epidemic at . . . xviii 369

ague at . . xii 558

population of . . . xvi 558

Tela vesicatoria of Messrs. Smith . xiii 64

Telangiectasis, or erectile tumour . xix 540

Teleaugiectasia lipomatodes . . ii 261

Teleological school, remarks on the v 89

Teleologist, reasonings of a . . v 89

Telluric spirit of magnetism . . vii 320

Temper, mania of, described . xx 28

observation on . . . xv 426

Temperament, and religion . xv 426, 429

bilious or choleric . . . xv 424

lymphatic . . . . xv 425

nervous . . . . xv 426

sanguineous . . . . xv 424

Temperaments, compound . . xix 55

gaseous theory of . . . xxi 146

mental effects of . . . xv 424

observations on . . . xix 53

M. Royer Collard on . . xviii 153

Mr. Combe on . . . xv 57

Mr. Mayo on . . . v 384

the different kinds of . . xix 53-5

Temperance, and teetotalism . xxiv 515

effects of, in India . . xi 259

on an argument against . xvii 53

Temperance Convention, the World's, Proceedings of . . . xxiv 515

movement, origin and progress of xxiv 521

Temperance ships, remarks on . xxiv 531

societies, progress of . . v 404

Society, first European . . xxiv 521

Temple, magnetic . . . vii 338

Temperature, Dr. Dunglison on . i 346

effect of exercise on . . xii 147

extremes of, danger of . xxiv 98, 113

human, in hot climates . . xii 141

in health and disease . . vi 142

in relation to diseases . . xxiv 107

vegetables . . vii 176

various diseases . . . ii 246

its influence on contagion . i 378

influence of, on health . . xxiv 96

on life . . . . xix 182

wounds . . vii 251

most agreeable, in rooms . xix 11

of different animals . . xii 142

fishes, Dr. Davy on . . ii 217

insects . . . . iv 527

reptiles . . . . xii 143

the body, after death . . xii 147

in diseases . . i 240

earth and sea . . xxii 229

sea, Dr. Becker on . ii 520

uterus and vagina . vii 549

tolerance of changes of . . iii 352

use of, in diagnosis . . xii 146

Temperatures, table of . . . xix 562

Temporal artery, on opening the . xiii 338

bone, comparative anatomy of . vi 201

Temporo-maxillary anchylosis . xiii 527

Tenasserim, medical reports on . xv 1

Tenderness of abdomen, diagnosis in iii 262

Tendinous tissues, anatomy of . xix 256

blood-vessels of . viii 594

Tendo Achillis, division of iv 231, 539, v 285

M. Bouvier on section of viii 385, 405

section of in club-feet . iii 233

Tendon of biceps humeri, displaced xiv 63

posterior tibial, section of . xxi 184

Tendons, and muscles, division of . xiii 1

rupture of . . x 355

changes in reunion of . . viii 390

cicatrization of . . . vii 240

circulation in . . . viii 594

division of . . . . viii 384

bandages after . . xxi 331

hamstring, section of . . viii 596

knife for section of . . viii 391

physiology of divided . . v 550

process of union in divided . x 356

redivision of . . . . xiii 25

reunion of, by seton . . xviii 224

supply of blood to . . xiii 25

suture in divided . . x 355

Tenotomy, and myotomy, history of xvi 66

applied to club-foot . . xxi 182

history of . . . . v 551

non-division of sheath in . xiii 2

period of extension after . xiii 6

repetition of . . . . xiii 25, 26

subcutaneous, Dr. Phillips on xiii 1, 23

Tenotomy, use of apparatus before xxi 184

Tentamen medicum, in Prussian universities . . . . i 296

Tentorium, origin of the nerves of the vi 204

Teratology, error of M. St. Hilaire in xii 384

etymology of . . . . viii 2

St. Hilaire on . . . . viii 2

TERME and MONFALCON, MM., on foundlings . . . xiii 289, 293

Terms, Medical, Dictionary of . ii 508

Mr. Farr's New Medical xviii 198

Ternary and quaternary compounds xviii 519

TERRY, Mr., on suspended animation viii 596

Testament, an eccentric . . . x 138

TESTE, Dr., on animal magnetism . xix 428

Test, new docimastic . . . . xvi 149

Testes, and cerebellum, connexion of xvii 402

minute structure of the . . xv 280

Testicle, arrestment of, with hernia xvii 61

cancer of . . . . . xxii 306

chronic inflammation of . xvii 80

descent of the . . . xvii 57, xx 136

causes of failure of . . xvii 59

extirpation of . . . . xii 428

fungous, and hæmatocele, diagnosis of . . . . i 476

and scirrhous, diagnosis of i 474

castration for, when proper and not . . . i 477-8

diagnosis of . . . i 474

duration of . . . . i 472

growth of . . . . xvii 80

hard at first . . . . i 475

hæmatocele mistaken for . i 476

inguinal glands in . . i 473

scrotum rarely ulcerates in i 472

fungus of, cured by castration i 477

Dr. Baring on . . . i 469

its etiology . i 476

primary seat of . . i 472

treatment of . . . . xvii 80

gonorrhœal swelled, treatment of x 393

indurated, mercury in doubtful cases of . . . . . i 476

inflamed, compression in . ii 253

cure of by pressure . iii 516

inflammation of . . . . xvii 74

rheumatism of . . . . xiii 447

swelled, compression in . . xii 373

curious case of . . xii 372

from gonorrhœa . x 392, xii 371

in clap, causes of . . xii 371

morbid anatomy of . xii 372

prognosis of . . . . xii 373

tubercular disease of . . xvii 82

undescended, on virility in . xvii 61

wounded, without danger . xii 256

Testicles, causes of atrophy of . xvii 65

operation on, peritonitis after . xvi 385

rapid death from pulling . xvii 270

report on . . . . . xvii 270

wasting of, from inaction . xvii 65

wounds of . . . . . xvii 66

Testimonium maturitatis of Prussian physicians . . . . . i 296

Testimony in philosophical cases . xix 431

Testis, anatomy of . . . . xvii 57

cancer of, diagnosis of . . xxii 307

condition of undescended . xvii 59

descent of, into perineum . xvii 64

through femoral canal . xvii 65

diseases of, Mr. Curling on . xvii 55, 87

encysted hydrocele of . . xvii 72

fungous, on operating on . v 416

granular swelling of . . xvii 80

gubernaculum of, structure of . xxii 202

hernia of . . . . . xvii 80

hydrocele of tunica vaginalis of xvii 67

imperfect descent of, diagnosis of xvii 64

inflammation of, effects of . xvii 76

in infants . . . xvii 79

irritable, causes of . . . . xvii 84

symptoms of . . . xvii 83

treatment of . . . xvii 84

its structure and secretion . xx 296

neuralgia of . . . . . xvii 83

scrofulous, disease of, symptoms of xvii 82

treatment of . . . xvii 83

Sir A Cooper on . . . xii 515

his treatise on . . x 103

on diseased . . v 417

various diseases of . . . . xvii 83

Tetanic spasm, Dr. Wilson on . xvi 422

Tetanus, a muscle fever . . xvi 422

and trismus infantum . . ix 531

Bellingeri's views of . . v 226

carbonate of iron in . iv 478, vi 453

cases of . . . . v 226, xx 264

by Mr. Alcock . . vi 453

cause and treatment of . . xx 115

cured by moral impression (?) v 228

Dr. O'Beirne on . . . ii 595

on the seat of . . ii 595

Dr. Wilson's pathology of . xvi 422

endermic use of morphia in . v 230

essential nature of . . . iii 464

experiments on the source of . iii 32

females less subject to . . iii 463

increased irritability in . . xi 454

in Hayti, Dr. Smith on . . v 229

its relation to spinal cord . iii 32

Mr. Curling's divisions of . iii 464

treatise on . . . iii 463

or tonic spasm of diaphragm . xxiv 175

pathognomonic sign of . . ii 595

Prof. Romberg on . . . xvii 421

pure, acute treatment of . . iii 464

seat of, in spinal cord . . xi 457

successfully treated . . vii 559

tobacco enemata in . . ii 595

traumatic, case of, cured . xiv 254

cure of . . . . . xiv 230

pathology of . . . xvi 420

treated with colchicum . . v 229

treatment of . . . . xi 457

use of electricity in . . xxiii 406

Tetanus, use of Indian hemp in . x 227
tobacco clysters in. iv 223
wourali poison in . . . viii 598
Texas, account of yellow fever in . xii 433
yellow fever, contagion in . xii 437
treatment of . . xii 437
TEXTOR, M., on regeneration of bone xviii 529
Textural anatomy, Burggraeve on . xxiii 136
Textures, permeability of, to gases . viii 322
remarks on . . . . xx 305
Thackeray prize . . . . x 591
Prize Essay, Dr. Davidson's
(App. to vol.) xi 1
THACKRAH, Mr., on health in trades xv 285
Thæria cavitatis nasi . . . xiii 467
orris . . . . xiii 466
faucium . . . . xiii 466
phlegmonosa, seu exedens . xiii 465
pustulosa area . . . xiii 464
figurata . . . . xiii 464
seu cutanea . . . xiii 463
tuberculosa, seu subcutanea . xiii 464
Thæria, corrosive sublimate, remedy for xiii 467
following other diseases . . xiii 463
of mucous membranes . . xiii 465
the external skin . . xiii 463
the osseous system . xiii 465, 467
or radesyge, description of . xiii 461
predisposing causes of . . xiii 467
premonitory symptoms of . xiii 462
Thalami optici, probable office of . xxii 310
THALES, philosophy of . . . v 82
Thallus-filaments in bronchial coagula xxiii 510
Theft, mania for, in pregnant women x 169
THEILE, M., on anatomy of the liver xxi 559
Theine, and tea, observations on . xvii 21
identity of, with caffeine . xiv 517
THEMISON, and his doctrine, account of xxiii 103
Theobromine, chemical properties of xiv 517
Theology, natural, remarks in relation to vii 521
THEOPHILUS, editions of his works xv 440
on the human body . . xv 439
some account of . . . xv 441
THEOPHRASTUS, genuine works of xiv 150
(Paracelsus) on his works . xiv 149
von Hohenheim . . xiv 148
Theories, and hypotheses, use of . xxiii 103
medical origin of . . . xxiii 97
observations on . . xxi 498
therapeutical, formation of . xxiii 105
Theory, influence of, on medical practice ii 5
on medicine . . xix 361
of disease, German . . xiv 328
Therapeutical theories, formation of xxiii 105
Liebig's . xv 501, 512
knowledge, remarks on . . xxii 236
Therapeutics, and hygiene, remarks on iii 458
and nature, in disease . . i 413
pathology, Dr. Marx on . xvi 472
physiology, relation of . xiv 197
on chemical . . . . xv 500
&c., Dr. Dunglison on . . xvii 242
Dr. James Arnott on . . xxi 498
Therapeutics, education in . . x 193
general, Schönlein on . . xxi 24
improvement of, remarks on . i 397
infantile . . . . . xx 551
report on . . . xxiii 299
in France, account of . . iii 449
Manual of . . . . xiv 140
of Van Helmont . . . xvi 416
ophthalmic, Rognetta's . . xxi 159
practical, on reform in . . xxi 202
present state of . . . xxi 257
remarks on British . . ix 465
Theriaki, or opium-eaters in Turkey iv 394
Thermal baths, use of, in paralysis . xiv 339
case, by Dr. Dickson . xv 126
comfort, Sir G. Lefevre on xv 541
Thermo-electric multiplier, use of . v 333
electricity, observations on xvii 448
Thermometer stove, Dr. Arnott's . v 546
Thermometers, dry and wet bulb . xxiv 283
Thermometrical table, Mr. Taylor's xix 562
Théroigne de Méricour, case of . vii 46
Theses, inaugural, remarks on . vi 148
medical, remarks on . . xv 314
THEVENOT, M., on Senegal . . xi 364, 382
THIELE, Dr., on cowpox and smallpox ix 76
THIERS, M., M. Bouillaud's retort to xxiv 262
Thirst, causes of . . . . xvii 42
Thigh, fractures of, new mode of treating xvi 508
Thigh-bone, head of, kept in socket by air ii 236
retention of . iv 205
Thinking force, or power, of Unzer xxiv 184
depends on fluid in the vessels ii 387
experiments on . . . ii 387
THOMANN, on nux vomica in dysentery i 256
THOMAS, Mr., his address . . vii 520
THOMPSON, Dr. Theophilus, his oration vi 196
on chorea . . . . xii 106-7
hysteria . . . . xii 107
influenza . . . . xii 111
THOMPSON, Mr. E., on the uvula . ix 219
THOMSON, Dr. A. S., on climate . vi 148
on fever . . . . vi 556
THOMSON, Dr. A. T., his Materia Medica
xvi 352, 359
on diseases of the skin . . x 250
poisonous iodides . . vi 585
the sick room . . . xi 511
THOMSON, Dr. John, Dr. Marx on . xv 20
THOMSON, Dr. R. D., on food . . xxiv 332
on indigestion . . . ix 273
nitrate of silver . . vi 581
respiration and animal heat xiv 297
the chemistry of digestion . iii 541
food of animals xxiii 157, xxiii 492
laws of cholera . . vi 576
THOMSON, Dr. T., his Organic Chemistry
xi 129, 151
on animal chemistry . xvii 424, 426
THOMSON, Dr. W., despondent practice of xii 88
black expectoration . iv 150, vii 154
diseases of the liver . . xii 78, 86
Thomsonian empirics in America . iii 276

Thoracic aneurism, case of . . viii 464
descending aorta, aneurism of . xxiv 416
disease, external signs of . iii 186
diseases, use of cold in . . ii 531
duct, analysis of its contents . xiv 537
aneurism of the . . ii 535
bifurcations of the . . iv 344
generally plexiform . iv 334
insensibility of . . iv 343
effusion, sublimate ointment of xxiv 45
three sub-stages of . xiii 365
fistula, remarkable case of . xiv 556
inflammation, pathology of . xx 79
organs of old women . . i 554
rachitis, queries on . . . v 139
viscera, on diseases of . . xix 21, 22
Thorax, and lungs, changes of, in the aged i 233
cylindrical form of . . . ix 332
increase of capacity of . . vii 362
inequality of sides of . . xix 106
pathology of . . . . . ix 484
THORNTON, Dr., on the inhalation of ether xxiii 549
Thought, effects of, on the brain . iv 437
mesmeric penetration of . . xix 448
power of controlling . . xi 95
Thoughts on Diseases, Dr. Seymour's xxiv 139
Throat, and larynx, on diseases of . xxiv 482
cut, case of, by Mr. Liston . v 465
recovery from . xi 459
singular case of . . iv 515
treatment of cases of . v 466
-cutting, curious case of . . vi 171
diseases of follicles of the . xxiv 484
follicular diseases of, cases of . xxiv 486-9
on a peculiar affection of . x 286
sore, clergymen's, remarks on . xviii 294
of clergymen . . . xxiv 484
spatula for displaying the . xxiv 502
swabbing of, with caustic . xxiv 499
Thrombus, fœtal, etiology of . . v 585
of child's head in labour . v 584
Thrush, a contagious disease . . xxiv 431
and aphthæ, colour of . . xxiv 423
a parasite . . . . . xxiv 426
causes of . . . . . xxiv 430
definition of . . . . . xxiv 423
description of the disease . xxiv 427
different stages of . . . . xxiv 429
eruptions of skin following . xxiv 435
general treatment of . . xxiv 433
habitat of . . . . . xxiv 425
in children, Dr. Berg on . . xxiv 422
Indian, history of . . . xii 516
M. Bosch on . . . xii 516
seat and causes of . . xii 516
treatment of . . . xii 516
local treatment of . . . xxiv 433
of infants . . . . . vii 82
-parasite, conditions of life of . xxiv 426
prevalence and prevention of . xxiv 432
Thumb, and index finger, spasms of xxi 332
brought in pocket, reunited . iv 257
case of division, and reunion of . iv 257
Thumb, dislocation of, case of . xvi 231
Thunny, large nerves in gills of the ii 217
THURNAM, Dr., his statistics of insanity xxii 349
THURNAM, Dr., on aneurism . xi 463
of the heart vii 149
Thymic asthma, an improper title . ii 240
cases of, by Dr. Kopp, &c. ii 237-8
Dr. Hirsch on . . ii 237
duration of . . . ii 239
history of . . . ii 238
nature of . . . ii 239
objections in regard to . . ii 240
pathognomonic symptoms of ii 239
prognosis in . . . ii 240
the same as laryngismus stridulus ii 240
treatment of . . . ii 240
Thymus gland, a cause of croup . ii 237
anatomy of . . . vi 218
development of . . xx 160
on the function of . . xx 165-7
growth and decay of . xx 161
minute structure of . xx 162
Mr. Simon on the . . xxi 566
Sir A. Cooper on the . x 103
treatise on . . . xx 159
weight of . . . ii 239
Thyroid arteries, on tying the . v 463
artery, superior, tying . xxii 50
body, a diverticulum of blood xxiii 143
foramen, hernia of the . xiv 556
gland, carcinoma of . . xx 85
cure of dropsy of . vii 586
dropsy of . . . iv 478
Mr. Simon on . xxi 567, 569
Sir A. Cooper and Mr. King on iii 153
Tiberius, Emperor, killed by burking ix 50
Tibia, the, chronic abscess of . . xxii 172
division of, deformed . . ix 389
on excision of head of . . iii 130
shot through, without splitting xv 401
Tibial artery, posterior ligature of . xxiv 323
Tic douloureux, change of air in . xviii 58
continued narcotising in . xvii 411
cured, case of . . . iii 230
by salicine, case of i 572
description of . . . xviii 57
division of . . . xviii 58
Dr. Allnatt on . xii 524, xvi 511
Dr. Hunt on . . . xviii 56
during twenty-five years . xvii 410
from anemia . . . xviii 63
dyspepsia . . xviii 59
the spine . . xviii 63
tumour, case of . xiv 549
uterine disorder . xviii 64
Mr. Hutchinson on . . vi 511
observations on . . xx 264
periodicity of . . . xviii 57
poultice for . . . xviii 68
premonition of . . xviii 57
Prof. Romberg on . . xvii 410
Sir B. Brodie on . . xxii 170
Tic, from diseases of the brain . xviii 65
disordered liver . . xviii 62

Tic, from local mechanical causes . xviii 65
dyspeptic, on the diet in . xviii 61
treatment of . . . xviii 60, 62
use of arsenic in . . xviii 60
with visceral congestion . xviii 62
in a neuralgic habit . . xviii 58
Sir C. Bell's remedy for, remarks on vi 169
spasmodic, Dr. Hall on . . xiii 158
TICE, Mr., his case of bronchial calculus xviii 365
Tickling the fauces, vomiting by . xv 72, 71
TICKNER, Dr., his physiology of living
v 381, 409
TIEDEMANN, Prof., comparative physiology of iv 5
his experiments on the breath i 244
partiality on the negro . viii 384
on Duverney's glands . . xvi 155
oxalic acid as a poison . ii 284
pulmonary exhalation . i 242
the brain of the negro . iv 529
negro brain . . . v 585, 588
brain of negroes . . viii 374
breath . . . . i 244-5
watery vapour from the lungs i 242
Tight lacing, in women, mischief of xi 425
observations on . . xix 107
royal discountenance of . xix 107
Timber, kyanized, danger from use of xi 532
Time, in reference to physical signs iv 297
our estimate of the lapse of . xxii 165
revolutions of, in diseases . xxiii 588
Timid practitioner, description of the xxii 122
Tin, hydrochlorate of, medicinal use of vi 530
Tincture of aconite, dose, &c., of . xx 468
broom-seed, preparation of i 534
Tinea capitis, treatment of, various authors on
i 578
favosa, caustic iodine in . . xiii 245
new mode of treating . xiii 244
nitrate of mercury in . xiii 245
new etiology of . . . xiii 233
vegetable parasites in . . xiii 233
TINNION, Dr., his new theory of disease xv 540
Tinnitus aurium, Dr. Kramer on . xxiv 28
Tisan, la, de Feltz, recommended . vi 372
Tissue, adipose, Dr. Geddings on . i 153
continued nutrition of . . xv 278
Tissues, anatomy and physiology of xix 256,
xxi 546
animal, remarks on . . i 395
dead, Dr. Prout on nutrition from xvi 485
development of, report on xix 589, xxii 266
fibrous, regeneration of . . xvi 504
Liebig on the changes of . xiv 307
metamorphosis of, Liebig on . xiv 511
microscopical varieties of . ix 509
non-vascular, Mr. Toynbee on . xii 572
opposite polarity of . . xxiv 133
origin of . . . . . xv 278
pathology of . . . . . xiv 544
primary, of vegetables . . iv 6, 7
Schwann's classification of . ix 509
source of reproduction of . xviii 102
Tissus lardaces, M. Lisfranc on . xv 43
Title-page of an unknown genius xxii 547
Title-pages of books, on voluminous vi 98
Toad, on poison secreted by the . xii 136
Tobacco, deplorable effects from . xix 28
diatribe against . . . xi 340
Dr. Ticknor on the use of . v 411
dyspepsia, from the use of . xix 27
essay on . . . . . xi 512
externally, good effects of . viii 582
follicular disease caused by xxiv 494, 506
its effects on workmen of it . v 451
Spanish, its use in ozæna . i 270
Tobacco-clysters in tetanus . . ix 223
-enema, antidote for . . iv 114
Dr. Abercrombie on . . iv 114
in ileus and enteritis . . iv 114
poisoning by . . . xii 562
properties and use of . . iv 114
-enemata in hernia, dangerous . xv 41
tetanus . . . ii 595
-leaf on epigastrium, emetic . xv 73
-smokers, valuable hint to . xiii 63
-smoking, Dr. Marx on . . xv 24
follicular disease from xxiv 494, 506
Tobago, life and death in . . xxiv 530
TODD and BOWMAN, their anatomy, &c. xv 537
physiology xx 201
TODD, Dr. R. B., case of dissecting
aneurism by . . . xx 94
his Anatomy of the Brain, &c. xxi 452
Cyclopædia of Anatomy, &c. i 536
obligations to other writers xvi 463
preparations of frogs . . xviii 74
on gout and rheumatism xvi 460, 471
hearing . . . . . viii 68, 70
structure of the stomach . ix 565
TODD, Dr. T. J., obituary of . . x 599
physiological discoveries by . iv 281
Toe, deformed great, contrivance for iii 152
Toes, neuralgia between . . xix 561
pain of, from hemorrhoids . iv 133
prominent ulcer after loss of . xxi 331
ulcers between, treatment of . xiii 542
Tolerance of emetic tartar, remarks on i 418, 422
Toltecan family, history of the . x 476
people, skulls of the . . x 479
Toltecans, physiognomy of the, *fig.* x 481
TÖLTENYI, his Essay on Medical Theory xix 361
TOMES, Mr., on osseous tissue . xxiv 386
on the teeth . . . viii 158, 159
TOMMASINI, on Rasori's doctrine . vii 424
Sir J. Clark's letters to . . xv 529
Tone of the muscles, observations on xix 306
Tongue, the, cancer of . . . xxii 294
case of cramp of . . . . x 557
fatal wound of . . vi 69
comparative anatomy of . . xix 586
diagnosis in insanity from . iii 224
division of muscles of . . xvi 68
Dr. E. Williams on . . xviii 236
Dr. Ridge, on diagnosis by . xvii 524
dryness of . . . . . vi 138
fur on the, nature of . . v 138
gland in the top of . . xxi 281
glands of, report on . . xxi 558

Tongue, hairs growing on . . xv 235

in affection of the stomach . xix 26

incision of, in stammering . xii 210

in death, by strangulation . ii 419

its relation to gastric disease . ix 474

sympathy with stomach . vi 176

malignant disease of . . ix 433

muscles of . . . . . xiv 377

papillæ of, as organs of taste . ii 433

M. Burggraeve on . xxiii 145

peculiar, in cerebral disorder . v 402

state of, as a diagnostic . . ix 475

in various diseases . vi 138

structure of . . . . . vii 239

papillæ of . xxii 288

Tonicity, an element of diseases . xvii 476

Dr. Williams on . . . xvii 476

defective, remedies in . . xvii 477

Dr. Williams on . . xvii 477

excess of, Dr. Williams on . xvii 477

Tonic effects of cold bathing . . xxii 449

Tonics, after courses of medicines . xvii 480

and excitants, Rognetta on . xxi 160

in insanity, Dr. Guislain on . iii 223

ophthalmic diseases . . xi 56

with astringents . . . xvi 200

purgatives, use of . . v 391

Tonsils, case of stone in the . . xi 528

chronic enlargement of, in children xvii 558

Dr. Smith on extirpation of . i 267

excision of, in stammering . xii 210

follicular disease of . . xxiv 489

in relation to hearing . . viii 97

on removal of enlarged . . v 465

scissors for, *plate of* . . i 268

Toothache, use of aconite in . . xx 467

Tooth-germs, abortive, in ruminants xxi 68

-powders, generally hurtful . ii 373

Topaze frigate, on cholera from the xxii 328

Tophaceous concretions, origin of . xiii 264

secretions, in rheumatism vi 371, 373

remedy for . . xiii 264

Töplitz, mineral waters of . . xiv 351

Topography, medical . . . vi 1

of Berlin . . xxi 205

Torpedo, the electrical, organs of . xii 130

electricity, and structure of . xxiii 398

experiments on . . . xii 129

Torpor of skin, singular case of . iv 120

Torquay, as a winter residence . xiv 477

the water at . . . . xiv 471

Torre, Della, his microscopy . xiv 486

Torrid zone, duration of life in the . vi 149

Torsion, and ligature of arteries . xxi 287

of arteries, Prof. Porta on . xxii 101

Torticollis, new species of . . xi 250

or stiff neck . . . . vi 355

Tortoise, colossal, fossil remains of . xxi 476

Tortoises, lymphatic hearts of . x 256

temperature of . . . xii 143

Tortual on infantile diseases . ii 358

Torula cerevisiæ, or yeast plant . ix 579

Total abstinence, hard labour sustained in xxiv 540

Total abstinence, in a leaky ship . xxiv 538

medical attention to . xxiv 515

movement, origin of xxiv 520, 522

on sudden or gradual . xxiv 547

unadvisable . . . ii 388

Touch, curious change in sense of . iv 380

Toulmouche, M., on articulations of ribs . . . . xxi 124

Tours, the, in foundling hospitals xiii 292, 300

in relation to infanticide xiii 300

suppression of, in France xiii 302

Towne, Mr., on the incubated egg ix 396

Town and country houses, remarks on i 354

longevity in . xviii 199

Towns, and country, mortality in . iii 50, ix 357-59, xviii 204

deaths in, algebraic account of xviii 204

drainage of, remarks on . . i 354

report on . . . xviii 494

Dr. Guy on the health of . xxiii 240

health of, report on . xv 285, 304

large, fewer recruits from . xv 307

medical officer of inquiry for xxi 346, 349

miasmata of, ill effects of . xviii 502

refuse of, money-value of . xv 334

application of . xviii 495

report of commissioners on xviii 492-93

sanitary state of, report on . xxi 333

supply of water to . xviii 497, xxi 342

Townsend, Col., his story doubted . i 440

Townshend, Rev. Mr., on mesmerism xix 429, 474

Toxicology, Barzellotti on . . ix 55

on investigators in . . ix 59

report on the progress of . xviii 533

Toynbee, Mr., on causes of scrofula xviii 500

on diseases of the ear . . xiv 62

non-vascular tissues . . xii 572

pathology of the ear . . xviii 370

the human kidney . . xxiv 331

Tracey, Count de, on the art of teaching i 361

Trachea, and bronchi, tuberculosis of xv 86

and larynx, on wounds of . v 465

case of stricture of . . xv 153

changed secretions of . . v 429

dilatation of . . . . . xv 84

false membranes in . . v 429

fistula of, operation for closing vii 411

mulberry-like excrescence in . xxii 468

foreign bodies in . . . v 466

narrowed, heart-disease from . xvi 327

openings in, treatment of . xxi 312

or air-tubes, of insects . . vii 170

rupture of the, case of . . xiv 549

worms in . . . . . ii 249

Tracheitis and croup, difference of xii 109

Tracheotomy, case of . . . xi 452

cases requiring . . iii 265, v 466

diseases requiring . . . xxi 488

early, in laryngitis . . . xviii 291

history of . . . . . xvi 51

in croup . . . . . xiii 248

remarks on . . . xv 327

Tracheotomy, in intoxication . . iv 140
in laryngitis . . . . . xx 91
M. Trousseau's operation of . xvii 300
observations on . . . v 441
on an oval opening in . . v 466
report on . . . . xvii 557
Sir B. Brodie's case of . . xviii 370
statistics of . . . . . viii 558
successful case of xi 526, xiii 565, xiv 255
success of, in croup . . xii 110
Traction, magnetic, remarks on . xix 480
Trades, &c., effects of, on health . xv 285
Tradesmen, health of, comparative xx 424
TRAILL, Dr., his Medical Jurisprudence iii 492
Training and racing, remarks on v 151-52
Traits, diagnostic, in children . ii 356
Trance, hysteric, cases of . . xix 437
Tranchina, on bodies embalmed by v 221
Transactions, of Medical Society, Bombay xxi 373
of Medico-Chirurgical . ii 158, iii 1,
iv 138, vii 140, ix 431, xiv 49,
xv 147, xviii 364, xxiii 410, xxiv 321
of Provincial Association i 529, iii 159,
iv 477, vi 1, 380, ix 216, xiv 396,
xvii 92, xxii 319
of the Royal Society . . ii 212
of the Vienna Society . . xix 360
Transformation of forces illustrated xvii 449
Transformations, osseous, Dr. Gross on xxiii 68
Transfusion in uterine hemorrhage xi 260
of blood, experiments on . vii 548
Trans-Himalayan region . . viii 117
Transition of disease into another . xxiv 319
on discerning . iv 307
Translators and translations, remarks on iii 488
Transmission, hereditary, of diseases xxi 417
Transpiration, daily and hourly . xix 568
Transplantation of skin . . . vii 394
Transposed viscera. fatal case of . iv 479
Transylvania, (U. S.), Medical Journal iii 195
University . ii 588
TRANT, Mr., his instrument for artificial anus . . . . . xxiii 27
Traumatic inflammations, bleeding in xiv 5
ophthalmia, Dr. Schindler on ii 267
Travelling, injurious in melancholia xxiv 153
Physician, Life of a . . xvi 265
physicians, institution of . xvi 459
TRAVERS, Mr., a curious illustration by ii 8
case of ruptured hernia by . xi 448
estimate of his writings . . ii 32
his case of tracheotomy . . xi 452
Hunterian Oration . . vi 191
introductory lecture . . i 209
on constitutional irritation . ii 1, iii 1
dislocated thigh-bone . iv 143
hydrocele . . . . . iii 560
inflammation . . . xviii 69
nervous influence . . iii 2
prostration . . . xxiii 559
removal of clavicle by . . vii 147
Treatment, and history, of diseases xxi 501
medical, Dr. Canstatt on . xiii 336
Treeak farook, account of . . v 129
Treeak farook, an Indian medicine . v 129
Trees, experiments on the heat of . xi 396
remarks on rearing and cutting down i 353
Tremor, nervous, of children . . vii 583
Trephine, Chelius on the use of the xx 116
Trephined old woman, case of . xiii 132
Trephining, observations on . . v 459
history of . . . . xvi 47
operation of . . . . xiii 134
paralysis cured by . . . xv 175
the skull, case of . . . v 460
TREVIRANUS, Prof., his botany . iv 1, 3
his microscopy . . . xiv 488
obituary and works of . . v 313
on structure of the retina . vi 394
Trichiasis, treatment of . . xviii 413
Trichina spiralis, observations on . xx 415
Trichocephalus dispar, frequency of ix 442
or trichuris v 594
Trichuris, Dr. Bellingham on . v 594
Tricuspid, its action as a safety-valve iv 364
valve in heart described . . iv 366
TRIER, Dr., his work on auscultation, &c. iii 189
Trigeminus nerve, irritation of . xi 230
Trigla (gurnard), spinal cord of . v 495
Tripe de roche of arctic hunters . xvii 20
Triplets, case of . . . . xiv 583
history of a case of . . xix 369
in 16,434 labours . . . ii 72
Trismus, from pericarditis, case of . ix 432
in newborn infants . . x 275
Trismus infantum, cause of, in West Indies ix 532
nascentium, in Vestmann Isles viii 486
Dr. Clarke on . . ii 97
report on . . xxiii 300
neonatorum, cause of . . xiii 528
epidemic . . . xiii 249
report on . . . xx 552
Triticum repens, medical use of . xix 241
Trocar for paracentesis thoracis, new xviii 344
Trombo, the, in arteries after ligature xxii 94
Troops, black, fatality of phthisis in vi 150
in Africa, mortality in . vi 150
British, on sickness, &c., of . ix 63
in Great Britain, health of . viii 223
in West Indies, mortality of vi 150, viii 211
quartering of, in West Indies xxi 174
statistics of health of British . viii 211
Trophical or organic nerves . . xvii 409
Tropical climates, and diseases . xiii 347
diet in . . . xiii 352
Dr. Johnson on xiii 347, 364
disease, mental shield from . xiii 352
diseased livers, theory of . xx 343
Tropics, comparative mortality in . vi 150
diet in, Mr. Jeffreys on . xvii 153
TROUSSEAU, M., on diseases of the larynx . . . . v 424, 428
on tracheotomy in croup . xvii 299
TRUMAN, Dr., on food and aliments xvii 1, 54
Truss, for reducible crural hernia . xxii 207
Mr. England's, for umbilical hernia . . . . xxii 208
new, for radical cure of hernia iii 234

Trusses, evils of high-pressure . vi 343-44

- for hernia, Mr. Teale on . . xxii 204

  - remarks on . . xiv 101

  - with solid blocks . v 301, 306

- hernial, faults of common . viii 561-63

  - M. Malgaigne on . . xiv 104

  - radical cures by . . xiv 104

- in hernia, axiom on the use of vi 343

- use and effect of . . . vi 343

Truth, and error, on admixture of . xix 429
- natural and divine, connexion of v 548

Truthfulness in medical writings .

Truths, living, Mr. Green on . . xxiv 220

Tschechi of Bohemia, remarks on . xviii 379

TSCHETERKIN, Dr., on purulent ophthalmia . . . ii 501

TSCHUDI, Dr., on Peruvian skulls xviii 372

Tubal pregnancy, case of, with no signs iii 245
- ending in rupture . . iii 245

Tube, Eustachian, Guyot's invention for iii 80
- water moving through, sounds of v 590

Tubercle, an amorphous product . iii 378

- anatomy of, Dr. Lebert on . xxii 26

- and bronchitis, Dr. Otto on . ix 217

  - gray granulation, identity of xiv 185

  - scrofula, on identity of ii 597, xiii 339-40

- caco-plastic nature of . . xvii 498

- chemical constitution of . xiii 340

- consists of fibrin . . . xvi 147

- constituents of . . . xxiii 434

- crude, description of . . xxiii 430

- diseases incompatible with . xiii 340

- cyst surrounding . . . xiii 340

- Dr. Campbell on the origin of xiv 184

- Dr. Graves on pathology of . xvi 249

- erroneously called tissue . iii 378

- essential character of . . xvii 96

- expectorated . . . . xiv 241

- experiments on . . . xiv 185

- fact in relation to . . . vi 135

- formation, and nature of . xxiv 136

  - of, theories on . . xiii 341

- from thoracic inflammations . ix 325

- in children, report on . . xvii 565

  - different organs, table of . xvii 566

  - relation to inflammation . iv 319

  - the bladder . . . xv 103

  - the bronchial glands . . xvii 306

  - vertebral disease, forms of . i 575

- its appearance and consistence iii 378

- its seat in the air-cells . . iv 319

- Laennec on the nature of , iv 318-19

- microscopic appearance of . xiii 340

  - structure of . . . xxiii 515

  - views of . . . . xiv 240

- miliary, nature of . . . xxiii 430

- morbid anatomy of . . i 123

- M. Andral's notes on . . iv 318-19

- M. Kuhn's discoveries in . iv 318

- Mr. Carmichael's errors on . iii 379

- Mr. Mayo's opinion of . . iii 64

- not an entozoon . . . iii 378

Tubercle, not caused by inflammation iii 382
- of brain in children . xv 152, xvii 552
  - lungs, and lepra, analogy of xvii 96
    - extremely common . xvii 96
    - with emphysema . xi 410
- origin, &c., of . . . vii 553
- pathology of . . . . xx 218
- production of, from pus and saliva xx 123
- pulmonary, Dr. Addison on . xxiii 422
- reabsorption of . . . xxiii 437
- seat of, in cases of children . xx 97
- scrofulous, characters of . . xxiii 514
  - the origin of Pott's disease i 575
- softening of . . . . xxiii 434
- special vitality of . . . iv 318
- structure of, Mr. Addison on . xvii 95
- syphilitic, characters of . . xxiii 357
  - treatment of . . . x 415
- tissue of, in the sputa . . ix 333
- unorganizability of . . xvii 96
- vitality of . . . . iii 380
- vascularity of . . . xxiii 515
- yellow, and gray granulation xvi 430, 435

Tubercles, bronchophony in . . iv 321
- cerebral, in children . . vii 576
- discussion on the nature of . ii 598
- Dr. Gross's opinions on . . xxiii 69
- Dr. Harrison on . . . iv 244
- Dr. Kingston on . . . iv 151
- dullness on percussion in . iv 321
- early detection of, by expiration i 482
- enlargement of . . . iv 216
- expansion of ribs opposite . ix 333
- experiments on . . . xii 531
- first in upper lobes of lungs . i 488
- formation, by mercury . . viii 323
- from error of digestion . . xiv 190
- general deposition of . . iv 320
- healing of . . . . iv 151, 152
- hemoptysis in relation to . ix 326
- hydrocephalus caused by . xii 229
- in air-cells of a bird . . . iv 244
  - phthisis, Dr. Evans on . xxii 86
  - serous membranes . . iv 53, 245
  - the bones . . . . xx 220
    - brain, cases of vi 200, ix 574, xiii 503
    - heart . . . . xx 225
      - case of . . . xvi 385
    - intestinal canal . . xxiii 432
    - larynx and fauces . xi 239
    - lungs, absorption of . ix 341
      - curative process in xv 91
      - Dr. Hodgkin on . xi 409
      - Dr. Stokes on . iv 318
      - metamorphoses of xv 90
    - peritoneum . . . iv 49
    - spine, their effects . i 575
      - paralysis from . i 576
    - uterus impeding labour . xii 558
- laws relating to . . . xii 348
- localities in relation to . . vi 22
- mechanical theory on . . xiv 188
- mode of increase of . . xxiii 433

Tubercles, M. L'Heritier on . . xvii 445

M. Rochoux on . . . i 238

Mr. Carmichael on . . ii 598

never caused by hydatids . iv 216

not essentially malignant . i 23

origin of . . . . . iv 151-52

prevented by ferruginous bread xii 531

primary forms of . . . i 238

red lines in . . . . . iv 152

relative frequency of, in organs xv 236

seat of, in the lungs . . xiv 186

Sebastian and Albers on . . iv 216

Sir J. Clark on the origin of . i 71

subcutaneous, on pain from . iii 25

variable consistence of . . iv 216

vascular formation of . . xiv 187

vascularity of . . iv 151, 216

why chiefly in the lungs . . xii 349

with emphysema . . . iv 321

Tubercular, and scrofulous, matter, on xxii 130

cavities in the chest . . xvii 307

cicatrization possible . . iv 217

deposit, M. Louis on . . xxii 136

deposits, external and internal ii 12

disease of brain . . . iii 315

spine, treatment of i 577

dissection of . i 575

infiltration, insular . . . xxiii 431

inflammations, M. Rayer on . ii 155

matter, the cells of . . xvii 445

constituents of . . xvii 446

fact on effusion of . . viii 192

Vogel on . . . xvii 445

meningitis in children . . xvi 445

perforations in the chest . xvii 306

phthisis, Dr. Gellerstedt on . xxiii 429

syphilides, proto-iodide of mercury in . . . . . xxiii 370

Tuberculated omentum, case of . iv 54

peritoneum, remarks on . iv 49, 53

Tuberculization, bronchial . . xvi 435

observations on . . . xxii 27

of lymphatic glands . . xvi 434

Tuberculo-pulmonic phthisis . . xxiii 421

Tuberculosis, and scrofulosis . . xiii 339

and typhus, analogy of . . xiv 243

cod-liver-oil gas in . . xiv 443

Dr. Gunsberg on . . . xxiv 127

of trachea and bronchi . . xv 85

primitive bronchial . . xv 86

Tuberculous affections, hereditariness of xxiii 519

the blood in . xvii 432

and scrofulous disease . . xxii 29

cachexia, Sir J. Clark on . i 72

climate for . . . xii 162

cavities, gray mass round . xvi 427

disease, causes of . . . xvi 452

cure of . . . xvi 452

diagnosis of . . . xvi 450

in children . . . xvii 304

diseases, essay on . . . iii 376

false membrane, in bronchi . xvi 429

impregnation of lungs . . xiv 189

Tuberculous infiltration of lungs . xv 90

lungs, purgatives in . . xiii 367

mass in pleura, case of . . iv 54

matter, characters of . . xvi 425

deposits of . . . xiii 339

in the blood . . . xiv 189

sputa . . . xvii 521

vertebræ . . xvi 385

primary seat of . . xvi 430

pulmonary products . . xxiv 127

Tuke, Mr., on hospitals for the insane xiii 213

on lunatic asylums . . ix 153

restraint of the insane . xiii 414

Tulloch, Major, his army reports xi 364, 381, xv 1, 18, xvii 313

on sickness, &c., of troops . ix 63, 76

the health of troops . viii 211

the statistics of negroes . iv 261

Tumeur de surprise, of Fournier . xiii 43

Tumour, abdominal, removal of, fatal ii 260

brain-like, of jaw . . . iv 147

cerebral, remarkable case of . xix 376

connected with liver . . xx 243

fibrous, mistaken for aneurism xvi 384

uterine, diagnosis of . vii 451

growth of, cause of intense pain in iii 24

in cauda equina . . . xiv 396

front of the retina . . xxii 31

pelvis, singular case of . xi 531

right hypochondrium, case of xx 99

lardaceous, on head, case of . viii 320

large congenital . . . xiv 583

malignant, case of . . . iv 479

of lip, hard, cured . . . xx 129

of the neck, peculiar . . ix 440

singular mesenteric . . xiv 126

uterine, 26 lbs. weight . . iv 373

Tumours, abdominal, Dr. Bright on vi 72, vii 462

adjacent to genital organs . xviii 34

after suckling a lamb, case of . xxii 157

bloody cranial, of infants . vii 86

of the scalp, in infants . i 182

deep, cautions on removing . xv 35

diagnosis of . . . . xv 33

right iliac . . . ix 454

discutient treatment of . . xiv 4

Dr. Lebert on . . . xxii 29

Dr. Warren on . . . v 414

on the origin of . . v 418

elementary development of . x 237

encysted, ossification of . . xviii 369

setons in . . . ix 441

erectile, cure of . . . ii 564

hemorrhoidal, case of . xxii 474

exploration of . . . xv 33

free incisions for . . . xv 35

general remarks on . . v 415

in brain, cases of . . . iv 376

neck, Mr. Phillips on . . xv 155

right iliac fossa . . . ix 446

indolent, origin of . . . ii 17

innocent and malignant . x 237

intra-thoracic, treatment of . xxii 218

Tumours, large, bisection of . . xv 35

vessel passing through . xv 35

ligature for portions of . . xv 36

Lisfranc on amputation of . xv 32

on extirpation of . . xv 32

malignant, diagnosis of . . xv 191

errors in removing . . xv 35

Vogel on . . . xxii 335

Mr. Macilwain on . . xx 127

non-malignant, Vogel on . xxii 334

of bone, pulsating . . xxiii 412

head and face, case of . xv 153

mouth, Mr. Liston on . iv 147

pelvis, congenital . . xiv 64

uterus, Mr. Lee on . . xxiv 506

on leeching . . . . . xiv 4

operations for . . v 415, xxi 291

osteoid, Prof. Müller on . . xix 537

over nerves and vessels . . xv 34

recto-vaginal . . . . . xviii 18

remarks on malignancy of . v 415

pendulous . . xv 34

removal of, Mr. Liston on . xxii 375

sero-cystic, of the breast . x 587

of female breasts . . xxii 167

solid, intra-throracic . . xxii 216

synovial, new treatment of . ix 554

tearing and stretching . . xv 35

the results of morbid irritation ii 17

treatment of . . . . xx 128-30

Tungusians and Manchew Tartars xxiv 462

Tunica Jacobi of the eye, remarks on the . . . . . ii 352

Ruyschiana of the eye . . x 12

vaginalis, condensation of . iv 173-4

hernia of . . . xxii 204

testis, indurated . . iv 172

treatment of indurated . iv 175

Tuphlo-enteritis, Dr. Burne on . ix 445

Türck, Dr., on spinal irritation . xix 96

Turgor vitalis, or active congestion iii 209

Turin, clinical medicine at . . ix 223

Turkey, account of diseases in . iv 392

women in . iv 391

a medical consultation in . iv 388

anatomy unknown in . . iv 382

boastful bonesetters in . . iv 396

Celsian lithotomy in . . iv 396

curious endemic disease in . iv 393

European physicians in . . iv 383

Frankish doctors in . . iv 383

great importance of the pulse in iv 387

prevalence of idiocy in . iv 391

Greek doctors in . . . iv 383-4

infrequency of mania in . . iv 390

Jewish doctors in . . . iv 383

lunatic asylums in . . iv 390

medical practice in . . iv 387

practitioners in . . iv 382-4

remuneration in . . iv 384

opium-eaters in . . . iv 394

peril of physicians in . . iv 389

physicians highly esteemed in . iv 384

poisoning in . . . . iv 389

Turkey, prevalence of scarlatina in . iv 392

smallpox inoculation in . . iv 392

spontaneous plague in . . xxiv 250

state of medicine in . iv 380, xv 282

surgery in . . iv 395

treatment of hernia in . . iv 396

vaccination in . . . iv 392

varieties of surgeons in . . iv 395

Turkish apparatus for fractures . vi 311

baths, account of . . iv 388

doctors, their white and black days iv 383

females, medical attendance on iv 385

harem, medical visit to a . iv 386

ignorance, &c., reflections on . iv 397

nation, change of skull in . xxiv 74

women, account of . . iv 391

Turks, barbarism and immorality of iv 381

general good health of . . iv 391

ignorant of the circulation . iv 382

talismans and amulets of . iv 382

their belief in the evil eye . iv 383

faith in astrology, &c. . iv 382

great medical ignorance . iv 381

ideas of diseases . . iv 382

Turley, Mr., on education . . x 243

Turn of life in women, remarks on the ii 513

Turnbull, Dr., apotheosis of . xv 495

meed of immortality to . . xv 494

most wonderful cases of . . xv 497-500

on diseases of the eye . . xv 494

on the ranunculaceæ . . ii 499

optical theories of . . . xv 495

Turner, Dr. E., biographical notice of iii 585

his Elements of Chemistry . v 216

his Chemistry, by Liebig . xxiii 584

Turner, Mr., on the astragalus . xviii 124

strictures on xviii 125-30

reply to his reclamation . . xviii 565

speech of . . . . . ii 605

Turner, Prof. J. W., of Edinburgh, his death . . . . . i 308

Turning in labour—Dr. Bush on . v 579

Dr. Churchill on . . viii 597

Dr. Dewees on bloodletting in difficult . . ii 79

Dr. Feist's remark on . iii 416

Dr. Kilian on . . . iii 417

Justina Siegmund on . iii 416

modes of . . . . xx 533

on informing the patient before xix 419

rupturing the membranes in iii 417, xiv 372

seizing the limbs in . xiv 372

remarks on . . iii 416, viii 48

Turning putrid children, Drs. Collins and Clarke on . . . ii 79

Turnip, constituents of the . . xvii 20

Turpentine, electuary of oil of . xiii 157

enemata in sciatica . . i 569

ethereal oil of, in typhus . xxii 473

in diseases of the eye . . vi 533

oil of, Martinet's formula of . xiii 157

Tuscany, maremma of, statistics of xxi 205

Tuson, Mr., on spinal curvature xii 177, 184

